# The tomato gene *Ty-6*, encoding DNA polymerase delta subunit 1, confers broad resistance to Geminiviruses

**DOI:** 10.1007/s00122-024-04803-w

**Published:** 2025-01-08

**Authors:** Xuexue Shen, Upinder Gill, Marjon Arens, Zhe Yan, Yuling Bai, Samuel F. Hutton, Anne-Marie A. Wolters

**Affiliations:** 1 Plant Breeding, Wageningen University and Research, Wageningen, The Netherlands; 2 Graduate School Experimental Plant Sciences, Wageningen University and Research, Wageningen, The Netherlands; 3 Department of Horticultural Sciences, Gulf Coast Research and Education Center, University of Florida, Wimauma, FL USA; 4 Present Address: KWS, Wageningen, The Netherlands; 5 Present Address: North Dakota State University, Fargo, ND USA

## Abstract

**Key message:**

The tomato *Ty-6* gene conferring resistance against begomoviruses has been cloned and shown to be a variant of DNA polymerase delta subunit 1.

**Abstract:**

*Ty-6* is a major resistance gene of tomato that provides resistance against monopartite and bipartite begomoviruses. The locus was previously mapped on chromosome 10, and in this study, we fine-mapped *Ty-6* to a region of 47 kb, including four annotated candidate genes. Via whole-genome resequencing of *Ty-6* breeding lines and several susceptible breeding lines, the polymorphisms in gene sequences were discovered and gene-associated markers were developed for marker-assistant breeding. Further, virus-induced gene silencing and candidate gene overexpressing in susceptible tomatoes revealed that *Ty-6-*mediated resistance is controlled by Solyc10g081250, encoding the DNA polymerase delta subunit 1, *SlPOLD1*. The single nucleotide polymorphism of *Ty-6* results in an amino acid change that might influence the fidelity of virus DNA replication.

**Supplementary Information:**

The online version contains supplementary material available at 10.1007/s00122-024-04803-w.

## Introduction

Geminiviruses are plant viruses that have either one or two circular single-stranded DNA molecules (2.5–5.2 kb), encapsulated in twinned (geminate) icosahedral particles (Fiallo-Olivé et al. 2021). Based on host range, transmission, phylogenies, and genome organization, geminiviruses have been classified into fourteen genera (ictv.global/taxonomy): *Becurtovirus*, *Begomovirus*, *Capulavirus*, *Curtovirus*, *Eragrovirus*, *Grablovirus*, *Mastrevirus*, *Topocuvirus, Turncurtovirus* (Zerbini et al. 2017), *Citlodavirus* (Fontenele et al. 2018), *Maldovirus* (Al Rwahnih et al. 2017), *Mulcrilevirus* (Lu et al. 2015; Ma et al. 2015; Qiu et al. 2020), *Opunvirus* (Fontenele et al. 2020), *Topilevirus* (Fontenele et al. 2017; Vaghi Medina et al. 2018). Most of the begomoviruses have bipartite genomes, DNA-A and DNA-B. Tomato yellow leaf curl virus (TYLCV) is a typical and well-characterized monopartite begomovirus. TYLCV ssDNA contains a non-coding intergenic region (IR) and at least six overlapping open reading frames (ORFs), which encode the capsid protein (CP)/V1 and movement protein (MP)/V2 in the viral-sense, and replication initiator protein (Rep)/C1, transcriptional activator protein (TrAP)/C2, replication enhancer protein (REn)/C3, and C4 in the complementary-sense. Recently, additional small ORFs were identified, of which the largest ORF was demonstrated to encode the V3 protein functioning as an RNA silencing suppressor and a viral MP (Gong et al. 2021, 2022). The intergenic region (IR) contains the origin of replication (*Ori*) and the promotors for V1, V2, C1, and C4. With limited coding capacity, TYLCV highly depends on the host molecular machinery to promote its infection.

TYLCV is transmitted by the whitefly *Bemisia tabaci* in nature and causes severe yield losses to global tomato (*Solanum lycopersicum* L.) production (Czosnek and Laterrot 1997; Varma and Malathi 2003). Tomato plants infected with TYLCV show typical leaf yellowing and curling, reduction in leaf size, stunting, as well as abortion of flowers and fruit. The most common strategy for managing tomato yellow leaf curl disease (TYLCD) is to apply insecticides to minimize vector populations. However, this method creates several issues, including pesticide resistance, damage to natural predators, and deleterious environmental and human health effects (Horowitz et al. 2007, 2011; Polston and Lapidot 2007; Vassiliou et al. 2011). Breeding for TYLCV-resistant cultivars is considered an effective and environmentally sustainable approach to reducing the loss caused by TYLCD.

Over the past two decades, multiple sources of resistance or tolerance to TYLCV have been identified in wild tomato species, including *S. chilense*, *S. habrochaites*, *S. peruvianum*, *S. cheesmaniae*, *S. arcanum*, *S. chmielewskii*, *S. corneliomulleri*, *S. galapagense*, *S. huaylasense*, *S. neorickii*, *S. pennellii*, and *S. pimpinellifolium* (Yan et al. 2018). Up till now, there are six resistance alleles/genes that have been identified and introgressed into cultivated tomato, with *Ty-1*, *Ty-3*, *Ty-4* from *S. chilense*; *Ty-2* from *S. habrochaites*; *ty-5* from *S. lycopersicum* cv. Tyking, and *Ty-6* presumed to originate from *S. chilense* (Anbinder et al. 2009; Gill et al. 2019; Hanson et al. 2000; Hutton et al. 2012; Ji et al. 2007, 2009; Lapidot et al. 2015; Scott et al. 2015; Zamir et al. 1994).

*Ty-1* and *Ty-3* are alleles of the same gene, which encodes an RNA-dependent RNA polymerase (RDR) that confers universal resistance against geminiviruses by enhancing the cytosine methylation of viral genomes (Butterbach et al. 2014; Verlaan et al. 2013; Voorburg et al. 2020). *Ty-2* codes for a nucleotide-binding domain and leucine-rich repeat-containing (NB-LRR) protein (Yamaguchi et al. 2018), which triggers a hypersensitive response (HR) in *Nicotiana benthamiana* by interacting with the viral effector protein Rep/C1 (Shen et al. 2020). As single dominant genes, *Ty-1*, *Ty-2*, and *Ty-3* are being used widely in breeding programs to introduce resistance to TYLCV. However, due to high recombination frequency in mixed virus infection (Díaz-Pendón et al. 2019; Fiallo-Olivé et al. 2019; Monci et al. 2002; Moriones et al. 2007) and error-prone replication (Ge et al. 2007; Yang et al. 2017), TYLCV variants have evolved rapidly and reduced the effectiveness of the resistance genes used in commercial cultivars. Breakdown of *Ty-1*-conferred resistance has been reported in Morocco, Italy, and Spain, with the occurrence of recombinants between TYLCV and Tomato yellow leaf curl Sardinia virus (TYLCSV) (Belabess et al. 2015; Granier et al. 2019; Panno et al. 2018). It is unclear how the recombinant strains break resistance. Given that the region of recombination was located in the intergenic region of the genome (IR) (Belabess et al. 2015; Panno et al. 2018), it might be related to the replication efficiency or viral gene expression. Resistance mediated by *Ty-2* also has been overcome by TYLCSV and the Mild strain of TYLCV (TYLCV-Mld) (Díaz-Pendón et al. 2019; Ohnishi et al. 2016). Fourteen amino acids in Rep/C1 (Avr determinant of *Ty-2*) have been found conserved in the resistance-breaking strains compared to resistance-inducing strains (Shen et al. 2020). However, which domain or amino acid residues of Rep/C1 determine the breaking of *Ty-2* resistance is still unknown.

The recessive gene *ty-5* encodes the tomato homolog of the messenger RNA surveillance factor Pelota (*Pelo*), which is involved in the ribosome recycling phase of protein synthesis (Lapidot et al. 2015). The T-to-G mutation in the first exon resulted in the loss of function of Pelota leading to resistance (Lapidot et al. 2015). *Pelo* is the first identified susceptibility (S) gene required for TYLCV infection. Loss-of-function of *Pelo* provides broad-spectrum resistance in different animal and plant species, such as to Drosophila C virus in Drosophila (Wu et al. 2014), to the bacterial pathogen *Xanthomonas oryzae* pv. *oryzae* in rice (Ding et al. 2018), to begomovirus isolates of PepYLCIV and PepYLCAV in pepper (Koeda et al. 2021). However, due to the recessive inheritance of *ty-5*, the exploitation of this resistance in breeding has been delayed.

Unlike the above-mentioned *Ty* genes, *Ty-4* and *Ty-6* are less characterized and have not been cloned yet. *Ty-4* was derived from the *S. chilense* accession LA1932 and mapped on the long arm of chromosome 3 (Ji et al. 2009). In contrast to about 60% variance explained by the *Ty-3* gene, *Ty-4* accounted for about 16% of the phenotypic variance (Ji et al. 2009). Thus, *Ty-4* had less effect on TYLCV resistance. Hutton et al. (2012) demonstrated the presence of a new resistance locus in the breeding lines Fla. 8638B and Fla. 8383, which was designated as *Ty-6* by Scott et al. (2015). More recently, *Ty-6* has been mapped on chromosome 10 and characterized as an incomplete dominant gene conferring intermediate resistance to monopartite begomovirus TYLCV and high resistance to bipartite begomovirus ToMoV (Gill et al. 2019). Moreover, *Ty-6* provides complementary resistance when combined with other resistance genes, like *Ty-3* and *ty-5* (Gill et al. 2019; Scott et al. 2015). Thus, it is likely that *Ty-6* will be very valuable in breeding programs. However, the exact gene and molecular mechanism underlying this resistance are still unknown.

In the present research, we fine-mapped *Ty-6* to a 47-kb region on the long arm of chromosome 10, which includes four annotated genes (ITAG4.1). Via overexpression and silencing of candidate genes in *S. lycopersicum* cv. Moneymaker and the *Ty-6* introgression line, the *Ty-6* gene has been identified as Solyc10g081250, a homolog of the Arabidopsis gene encoding the DNA polymerase delta subunit 1 (*POLD1*). Several different *POLD1* alleles in wild tomato accessions were identified.

## Materials and methods

### Plant materials and methods for experiments in Florida

#### Fine mapping

Fine mapping of *Ty-6* was conducted in a large-fruited, fresh market tomato population developed from the cross between Fla. 7804 and Fla. 8986. Fla. 7804 is a susceptible inbred line; Fla. 8696 is a begomovirus-resistant line based on *Ty-6*, which was derived from Fla. 8624 (Fig. S1A; Scott et al. 2015). Recombinants for initial fine mapping were identified at the F_2_ generation, and additional recombinants for further delimiting the *Ty-6* locus were identified among the F_3_ progeny of these.

### Marker development and recombinant screening

Lee et al. (2018) previously described whole genome re-sequencing of three *Ty-6* breeding lines (Fla. 8383, Fla. 8624 and Fla. 8638B; Fig. S1A) and of 16 susceptible breeding lines, including alignments to the ‘Heinz 1706’ reference genome for polymorphism discovery. Single nucleotide polymorphisms (SNPs) corresponding to the *Ty-6* region of chromosome 10 (Gill et al. 2019) and distinguishing between resistant and susceptible lines were identified and used for marker development (Table S1). Twenty SNPs were developed into Kompetitive Allele Specific PCR (KASP) markers and assayed by Ag-Biotech, Inc. Three SNPs were developed into markers for High Resolution Melting (HRM) analysis. The primers for HRM markers were designed using the IDT PrimerQuest tool (https://www.idtdna.com/PrimerQuest/ Home/Index), and the PCR protocol for these markers was as follows: Five µL PCR reactions were comprised of 0.5 µL of DNA solution (1–10 ng/ µL), 2 µL of AccuStart® II PCR SuperMix 2X (Quatabio, Beverly, MA), 0.25 µL of EvaGreen® Dye 2X (Biotium, Hayward, CA), 0.05 µL of each forward and reverse primers (0.1 mM), and 2.15 µL of molecular grade water. PCR amplification was performed using a Mastercycler® Pro 384 (Eppendorf, Hauppauge, NY) with the following temperatures and durations: 94 °C for 30 s for initial denaturation; followed by 40 cycles of denaturation at 94 °C for 5 s, annealing for 10 s (at 58 °C for HRM-5547–1; at 52 °C for HRM-6519; and at 53 °C for HRM-0239), and extension at 72 °C for 15 s; then a final extension at 72 °C for 1 min. HRM markers were detected by melting curve analysis using a LightCycler® 480 Instrument II (Roche, Pleasanton, CA).

For genotyping, DNA was extracted from young leaves of individual plants using a modified cetyltrimethylammonium bromide (CTAB) procedure (Fulton et al. 1995). Recombinants were initially identified from the fine mapping population using markers KASP_21951 and KASP_27552, which flank the *Ty-6* interval identified by Gill et al. (2019). Each recombinant was genotyped with the 20 KASP markers to determine points of crossing over. Recombinants were allowed to self-pollinate, and progeny were genotyped to identify individuals that were homozygous for the recombined *Ty-6* interval, which constituted recombinant inbred lines (RILs). A single plant from each RIL was planted to the field and used for producing seed of the fixed lines, and the remaining plants of the RILs were used for a TYLCV-inoculated trial in spring 2017. Additional recombinants within the *Ty-6* interval that were identified during RIL development were selected and used in the fall 2017 inoculated trial.

### Whitefly-mediated TYLCV inoculations

TYLCV inoculations were performed using viruliferous whiteflies according to the methods described by Griffiths and Scott (2001) with some modifications. Briefly, seedlings four weeks past the cotyledon stage (three to four leaves) were exposed to viruliferous whiteflies for one week in a growth chamber. For this purpose, a whitefly colony viruliferous for the Israeli TYLCV strain was maintained in temperature-controlled growth rooms (27 °C) on infected tomato plants, and whitefly-infested plants were transferred to a separate growth room for the inoculation. On average, 20 whiteflies per plant were used during the inoculations. Following inoculation, the whiteflies were killed by treating plants with insecticidal soap and with imidacloprid [Admire (Bayer CropScience, Research Triangle Park, NC)], prior to transplanting to the field.

Field trials were conducted in the spring and fall of 2017 and in the fall of 2022 and were arranged as randomized complete block designs, with two blocks and four-plant plots. Treatments in the spring 2017 trial included 50 RILs and each of the parents of the mapping population; the fall 2017 trial included parents and 12 select RILs; and the fall 2022 trial included parents and 25 select RILs. Plants were rated for disease severity approximately 40 days after exposure to whiteflies on a 0 to 4 disease severity index (DSI) scale as described previously (Scott et al. 1996), where 0 = no symptoms and 4 = severe symptoms and stunting. Intermediate scores such as 1.5, 2.5 etc. were incorporated to allow for more precise disease severity ratings.

### Identification of candidate genes

The sequences of the fine-mapped region were compared with the tomato reference genome (the Heinz genome SL2.50) and the genes annotated (ITAG3.2) in this region were identified as candidate genes. The amino acid sequences of the candidate genes were used in the BLASTP analysis in the Araport11 protein sequences datasets (https://www.arabidopsis.org/Blast/).

### Plant materials and methods for experiments in Wageningen

Breeding line Fla. 8383 (Gill et al. 2019) was used to generate the *Ty-6* homozygous introgression line PV123017 in the background of cultivated tomato species *S. lycopersicum*. Selfing progenies of PV123017 were used for the experiments conducted in Wageningen (Fig. S1B). *S. lycopersicum* cv. Moneymaker (MM) was included as a susceptible control. In VIGS experiments, the introgression line PV103208 carrying the resistance gene *Ty-1* was also included as a control. The plants were grown under greenhouse conditions (16 h light at 21 °C and 8 h dark at 19 °C), with 60% relative humidity.

### Time-course experiments and gene expression analysis

To investigate the transcriptional changes of the candidate genes in resistant plants carrying the *Ty-6* gene and susceptible MM plants in response to TYLCV infection, a time-course experiment was performed on *Ty-6* introgression line TV140124 in greenhouse conditions followed by RT-qPCR analysis. Thirty seedlings were infected with TYLCV, thirty were mock-infected, and five were not treated at all. MM was included as a negative control. The top leaves of three randomly chosen plants were collected at 1, 3, 7, 14, 21, and 28 days post inoculation (dpi). In addition, leaf samples were collected at 0 dpi from three non-inoculated plants. Total RNA was extracted using the RNeasy Plant Mini Kit (Qiagen). DNase I (Invitrogen) was used to remove the DNA contamination. RNA quality and concentration were determined using agarose gel electrophoresis and NanoDrop™ One (Thermo Scientific). Subsequently, cDNA was synthesized with the iScript™ cDNA Synthesis Kit (BIO-RAD). Quantitative RT-PCR was conducted in a Bio-Rad iCycler iQ5 using iQTM SYBR Green Supermix (BIO-RAD). The tomato *Ubiquitin* gene Solyc07g064130 was used as the endogenous control for normalization. The information on the primers used for gene expression analysis is shown in Table S2. Data were analyzed by the 2^−∆∆CT^ method (Livak and Schmittgen 2001). The relative expression levels of the target genes were normalized to that of *Ubiquitin*, and subsequently related to MM time point D0 (0 dpi).

### TRV-based virus-induced gene silencing (VIGS)

The TRV-based VIGS system (Liu et al. 2002) was used to transiently silence the candidate genes. Briefly, 150–300 bp fragments of the candidate genes were amplified using *Ty-6* cDNA as a template with specific primers (Table S2) and cloned into pENTR/dTOPO entry vector (Invitrogen). After colony PCR and sequencing, the plasmids carrying the amplified fragments were subcloned into the pTRV2 destination vector. All the constructs were transformed to *Agrobacterium* strain GV3101 for agroinfiltration. *Agrobacterium* cultures carrying the appropriate constructs were grown as described by Verlaan et al (2013). The OD_600_ of each culture was adjusted at 2. After 1–6 h of incubation at room temperature, pTRV2 cultures were mixed with pTRV1 culture at a 1:1 (vol/vol) ratio. The TRV infiltration was performed on cotyledons of 10-day-old seedlings using a needleless syringe. Plants infiltrated with TRV1 + TRV2:GUS were included as the control. Two weeks after TRV infiltration, plants were inoculated with TYLCV. To verify the silencing efficiency of the VIGS constructs, five TRV-infiltrated plants were mock-inoculated. The expression levels of the targeted genes were quantified three weeks after the TRV infiltration via RT-qPCR.

### RNAi, CRISPR/Cas9, overexpression constructs, and plant transformation

To generate RNAi constructs, the cloned fragments of the candidate genes as used for VIGS were recombined from the pENTR/dTOPO entry vector to the pHELLSGATE8 vector via LR Clonase reaction (Helliwell et al. 2002; Helliwell and Waterhouse 2003). The recombined plasmid was transformed to *E. coli* DH5α and subsequently transferred to *Agrobacterium* strain AGL1 carrying the *VirG* gene for plant transformation. Tomato transformation was conducted as described by Huibers et al. (2013) using cotyledons of seedlings of the *Ty-6* introgression line PV140124. Transgenic plants were identified by amplification of *NPTII* and CaMV-35S (Table S2) using Phire Plant Direct PCR Kit (Thermo Scientific).

To knock out the candidate genes, we generated CRISPR/Cas9 constructs containing four single guide RNAs (sgRNAs) targeting the first exon of each gene using Golden Gate cloning. The sgRNAs were designed using CRISPOR (Concordet and Haeussler 2018) (Table S2). The CRISPR/Cas9 constructs were built following the description by Belhaj et al. (2013), Brooks et al. (2014) and Weber et al. (2011). Briefly, the scaffold clones containing sgRNA were obtained by PCR using pICH86966 as a template. The PCR products and pICSL01009 (containing AtU6) were assembled into Level 1 vectors to generate Level 1 constructs (pICH47751::AtU6p::sgRNA1, pICH47761::AtU6p::sgRNA2, pICH47772::AtU6p::sgRNA3 and pICH47781::AtU6p::sgRNA4). The four Level 1 constructs, pICH47732 (containing *NPTII*), pICH47742 (containing *Cas9*), and the linker pICH41780 were ligated to Level 2 vector pAGM4723 and subsequently transformed to *E. coli* DH5α. The plasmid carrying all the components was selected and introduced to *Agrobacterium* strain AGL1 + *VirG*. Plant transformation was performed as mentioned previously. To select mutant transformants, the flanking regions of the sgRNA target sites were amplified by PCR using the specific primers (Table S2). Subsequently, the PCR products were analyzed by gel electrophoresis and sequencing.

To overexpress the candidate genes in the MM background, we constructed overexpression constructs carrying the full-length coding sequence of the candidate genes. First, the Maxima H Minus First Strand cDNA Synthesis Kit (Thermo Scientific) was used to synthesize cDNA with RNA of the *Ty-6* introgression line PV140122 (Fig. S1B) as template. The full-length coding sequences of the candidate genes were cloned from the synthesized cDNA using primer pairs G240-pD221-attB1 + G240-pD221-attB2 and G250-pD221-attB1 + G250-pD221-attB2 (Table S2). The coding sequences were recombined into the pDONR™221 vector (Invitrogen) via BP Clonase reaction and introduced into the destination vector pK7WG2 (Karimi et al. 2002) via LR Clonase reaction (Invitrogen). The plasmid containing the cloned coding sequence of the candidate genes was selected by sequencing and transformed to *Agrobacterium* strain AGL1 + *VirG*. The cotyledons of MM were used for transformation as described previously. Transgenic lines were identified by PCR amplification of *NPTII* and CaMV-35S (version in pK7WG2) (Table S2).

### Viral disease assays

For TYLCV disease assays, a TYLCV-IL-Alm clone (GenBank AJ489258), kindly provided by Dr. Eduardo Rodríguez Bejarano, Universidad de Malaga) was transformed into *Agrobacterium tumefaciens* strain LBA4404 (Morilla et al. 2005) and used for agroinoculation on three-week-old tomato seedlings as described by Verlaan et al. (2011). To test the resistance spectrum of the *Ty-6* gene, tomato yellow leaf curl Sardinia virus (TYLCSV) (GenBank X61153; kindly provided by Dr. Emmanuela Noris, Institute for Sustainable Plant Protection (IPSP), Torino, Italy), tomato yellow leaf curl virus—China:Shanghai2 isolate (TYLCV-[CN:SH2]) (GenBank AM282874; kindly provided by Prof. Bingyan Xie, Chinese Academy of Agricultural Sciences, Beijing, China) and Curtovirus beet curly top virus (BCTV) (GenBank M24597, kindly provided by Dr. Keith Saunders, John Innes Centre, Norwich, UK) were also included by Agrobacterium-mediated virus inoculation (Verlaan et al. 2011). *Agrobacterium* cultures containing TYLCV-IL-Alm, TYLCV-[CN:SH2] or BCTV were infiltrated at OD_600_ = 0.5, while culture containing TYLCSV was used for infiltration at OD_600_ = 1. The geminiviral symptoms of the inoculated plants were evaluated using a 0–4 disease severity index (DSI) score (Friedmann et al. 1998). Additionally, intermediate scores, 0.5, 1.5, 2.5, 3.5, were included in this study to provide a more accurate assessment of disease severity. After the last score, top young leaves were harvested and stored in a − 80 °C freezer for geminivirus detection and gene expression analysis.

### TYLCV DNA detection via quantitative PCR

DNA was isolated from leaf samples using CTAB following the protocol described by Untergasser (2008). The DNA concentration and quality were measured by NanoDrop™ One (Thermo Scientific). The quantification PCR of TYLCV DNA was performed using iQ™ SYBR Green Supermix (BIO-RAD) with primers TYLCV-IS 1678F and TYLCV-CONS 1756R (Powell et al. 2012). *Ubiquitin* Solyc07g064130 was used as a housekeeping gene. Primer sequences (Yang et al. 2014) are shown in Table S2. The relative DNA amounts of TYLCV were calculated using the 2^−∆∆CT^ method (Livak and Schmittgen 2001), where values were normalized against *Ubiquitin* and relative to the mean of the control plants.

### Statistical analysis

The statistical significance shown in figure legends was determined with a two-tailed student’s t-test or one-way ANOVA in GraphPad Prism software software or Microsoft Excel. P values less than 0.05 are considered to be statistically significant. The number of asterisks reflects the level of significance (* = *p* < 0.05; ** = *p* < 0.01; *** = *p* < 0.001; **** = *p* < 0.0001).

### POLD1 protein sequences analysis and allelic variation discovery

The protein domains of tomato POLD1 (Solyc10g081250) were analyzed using the NCBI’s CD-Search service (https://www.ncbi.nlm.nih.gov/Structure/cdd/wrpsb.cgi) against the database NCBI_Curated – 17,937 PSSMs (Lu et al. 2020). Non-synonymous SNP variants in tomato *POLD1* were identified using the whole genome resequencing data in 150 tomato variants browser (Wageningen UR 150 Tomato Genome Resequencing Project) (100 Tomato Genome Sequencing Consortium et al. 2014). The functional effect of the amino acid substitutions in *Ty-6* and other allelic variants on the protein was predicted using PANTHER (http://www.pantherdb.org/tools/csnpScoreForm.jsp) and PolyPhen-2 (http://genetics.bwh.harvard.edu/pph2/), accessed May 2, 2019.

## Results

### *Ty-6* was fine-mapped to approximately 54-kb region on chromosome 10

**Fig. 1:**
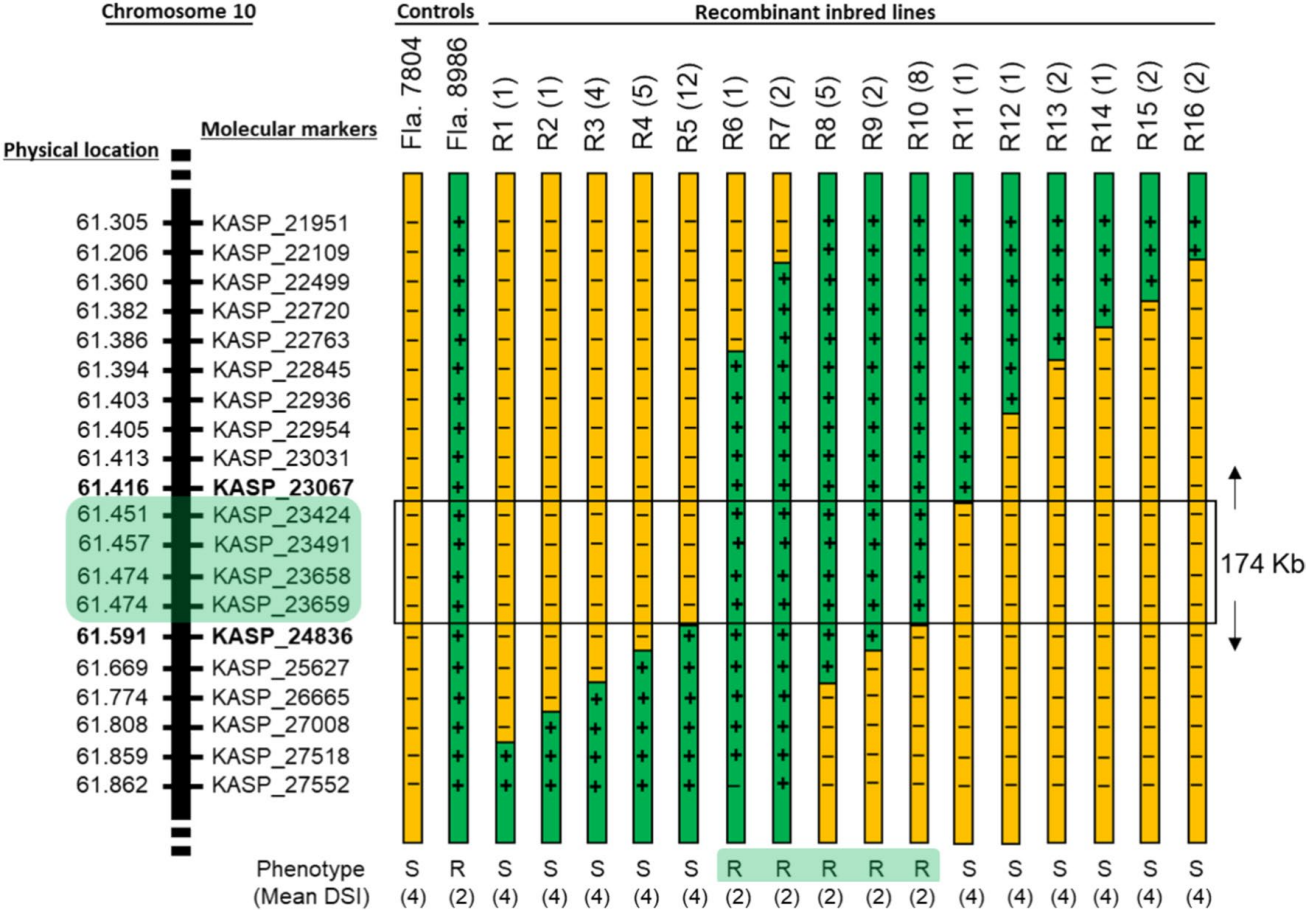
Fine mapping of *Ty-6* on chromosome 10. Recombinant breakpoints of 50 F_3_ recombinant inbred lines (RILs) among 20 KASP markers spanning a ~ 560 kb region on chromosome 10 of tomato. RILs within each of the 16 groups (R1 to R16) have the same marker genotypes, and the number of distinct RILs having the indicated genotype is indicated in parentheses (e.g. four RILs had the genotype indicated by R3, five RILs had the genotype indicated by R5, etc.). TYLCV disease reaction and mean disease severity index (DSI) of each recombinant group is provided along the bottom of the Figure. Chromosome segments representing the susceptible parent are shaded yellow (“-” represents homozygosity for the susceptible parent allele) and resistant parent genotypes are shaded green (“ + ” represents homozygosity for the resistant parent allele). Molecular markers and their physical positions are based on the SL4.0 tomato genome assembly available through the Sol Genomics Network (SGN; https://solgenomics.net)

We previously mapped *Ty-6* to chromosome 10 of tomato (Gill et al. 2019), and due to its importance in conferring resistance against monopartite and bipartite begomoviruses, we proceeded to fine-map this gene. A 700 plant F_2_ population from the cross between the *Ty-6* parent, Fla. 8986, and the susceptible parent, Fla. 7804, was used for recombinant screening. Markers KASP_21951 and KASP_27552 were used to identify 50 recombinants within the nearly 560-kb interval that these markers flank. Recombinant inbred lines (RILs) corresponding to each of the 50 recombinants were genotyped with 18 additional SNP markers that span the interval, and phenotyped in the field for TYLCV response. Results located *Ty-6* within a 174-kb physical region between markers KASP_23067 and KASP_24836 (Fig. 1).

**Fig. 2:**
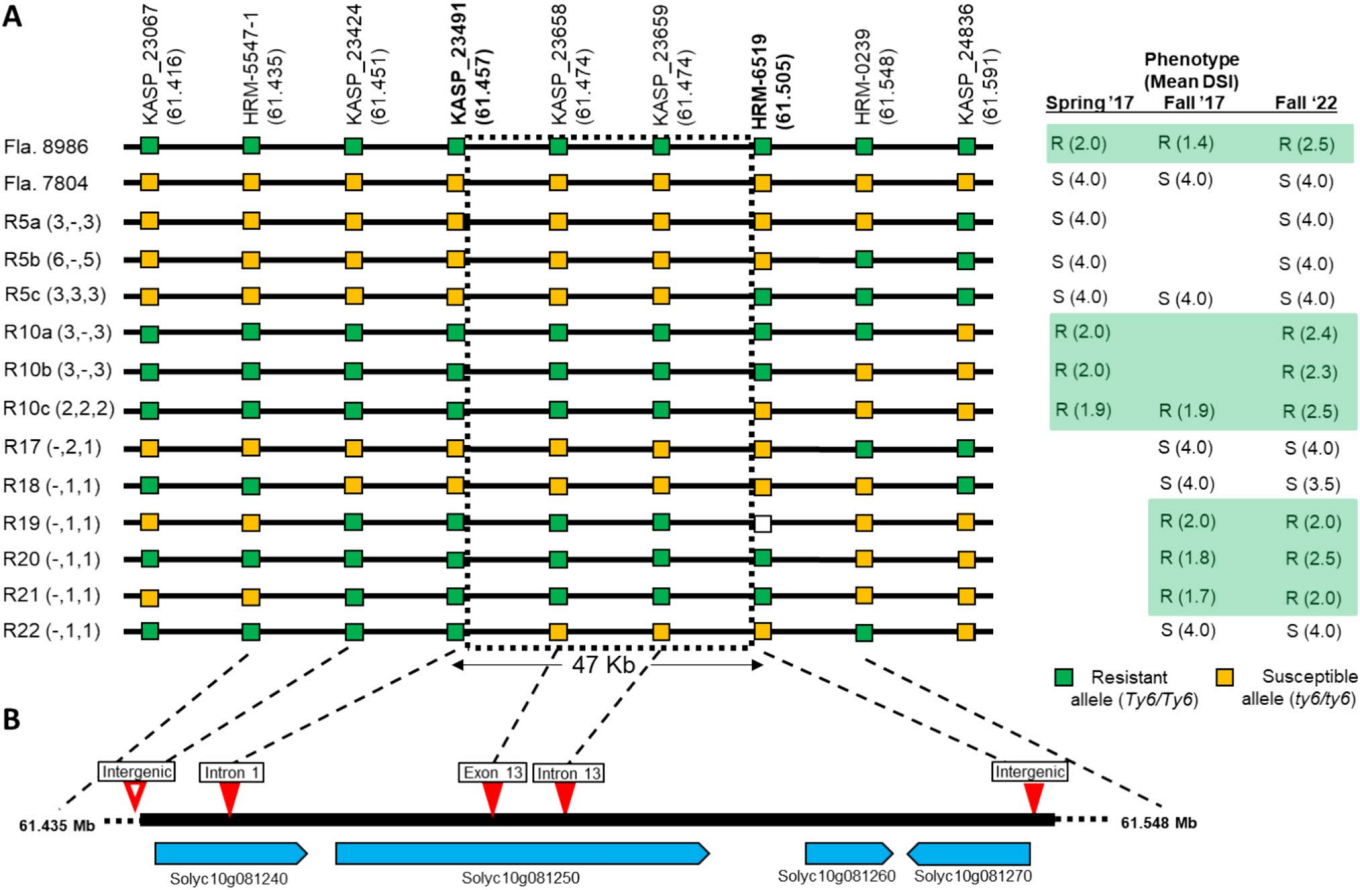
High resolution mapping of the *Ty-6* locus and identification of putative candidate genes. **A** The TYLCV disease reaction, mean disease severity index (DSI), and marker data of 27 key recombinants in 12 recombinant groups identifies a 47-kb physical region containing the *Ty-6* gene. The number of recombinants within each group and corresponding to each season, respectively, is indicated in the parentheses following each group number (for example, R5a was represented by 3 distinct recombinants in spring 2017, none in fall 2017, and three in fall 2022). At each marker, homozygous susceptible genotypes are shaded yellow, and homozygous resistant genotypes are shaded green; no shading indicates missing genotype data. **B** Locations within (solid red triangle) or adjacent to (open red triangle) the 47-kb *Ty-6* interval of five SNPs between resistant and susceptible parents and of two annotated genes that are *Ty-6* candidates. Molecular markers and their physical positions are based on the tomato genome assembly SL4.0 available through the Sol Genomics Network (SGN; http://solgenomics.net/)

To more precisely map *Ty-6*, three additional SNPs within this interval were developed into HRM markers and used to genotype the most informative recombinants. Furthermore, 1260 plants of a segregating population was screened to identify six new recombinants within the genic interval. Phenotypes of all these RILs were determined in fall 2017 and/or fall 2022 inoculated trials. Genotypic and phenotypic evaluation of these RILs reduced the *Ty-6* genomic interval to a 47-kb region demarcated by markers KASP_23491 and HRM_6519 (Fig. 2).

### Two out of four identified candidate genes showed polymorphism in gene sequences

Based on ITAG 4.1 gene models, the 47-kb fine mapped interval contains a portion of Solyc10g081240, as well as Solyc10g081250, Solyc10g081260 and Solyc10g081270, which encode GrpE protein, DNA polymerase POLD1, Protein DETOXIFICATION, and ELMO/CED-12 family protein (ITAG 4.1 annotation).

Polymorphisms in the candidate genes were detected by comparing whole-genome re-sequencing (Lee et al. 2018) alignments between resistant and susceptible inbred lines. Only five SNPs were discovered, and each of these were consistently polymorphic between resistant and susceptible lines. Three of these SNPs were located within genes Solyc10g081240 and Solyc10g081250, and no polymorphisms were found in the sequences of Solyc10g081260 or Solyc10g081270 (Fig. 2). Therefore, only Solyc10g081240 (GrpE protein) and Solyc10g081250 (DNA polymerase POLD1) were likely candidates for *Ty-6*. Two SNPs were identified in the genomic sequence of Solyc10g081240. The first, an A- > T SNP which corresponds to KASP_23424, is upstream of the gene in the non-coding region; this SNP is just outside of the 47-kb fine mapped interval. The second, a G- > A SNP which corresponds to KASP_23491, is in an intron, but the open-reading frame (ORF) of Solyc10g081240 is identical in resistant and susceptible parents (Table S1; Fig. 2). The two polymorphisms within Solyc10g081250 correspond to markers KASP_23659 and KASP_23658, respectively (Table S1; Figs. 1 and 2). They include a T- > G SNP in intron 13 and a non-synonymous T- > C SNP in exon 13 (Fig. S2A). Notably, the latter polymorphism results in the change of the 515th amino acid from Y (Tyrosine) to H (Histidine) (Y515H, Fig. S2B, S3) in the *Ty-6* lines. This is “probably damaging” to the function of the POLD1 protein encoded by the *Ty-6* allele, as predicted using SNP effect programs PANTHER and PolyPhen-2. Moreover, the T- > C SNP seems to be associated with the TYLCV disease response (Fig. S3B). Thus, the single amino acid difference in Solyc10g081250 makes it a strong candidate of *Ty-6*.

### Expression of the candidate genes after TYLCV infection

To investigate the expression pattern of Solyc10g081240 and Solyc10g081250 at several time points after TYLCV infection, we conducted a time-course experiment (Fig. S4). Solyc10g081240 expression was reduced in both MM and the *Ty-6* line at 7 days post inoculation (dpi), both in mock-treated and TYLCV-inoculated plants. Similarly, expression of Solyc10g081250 decreased after 7 dpi in both mock-treated and TYLCV-inoculated plants of the *Ty-6* line. In contrast, Solyc10g081250 expression increased after TYLCV infection in MM plants.

### Silencing Solyc10g081240 does not compromise TYLCV resistance in *Ty-6* plants

**Fig. 3:**
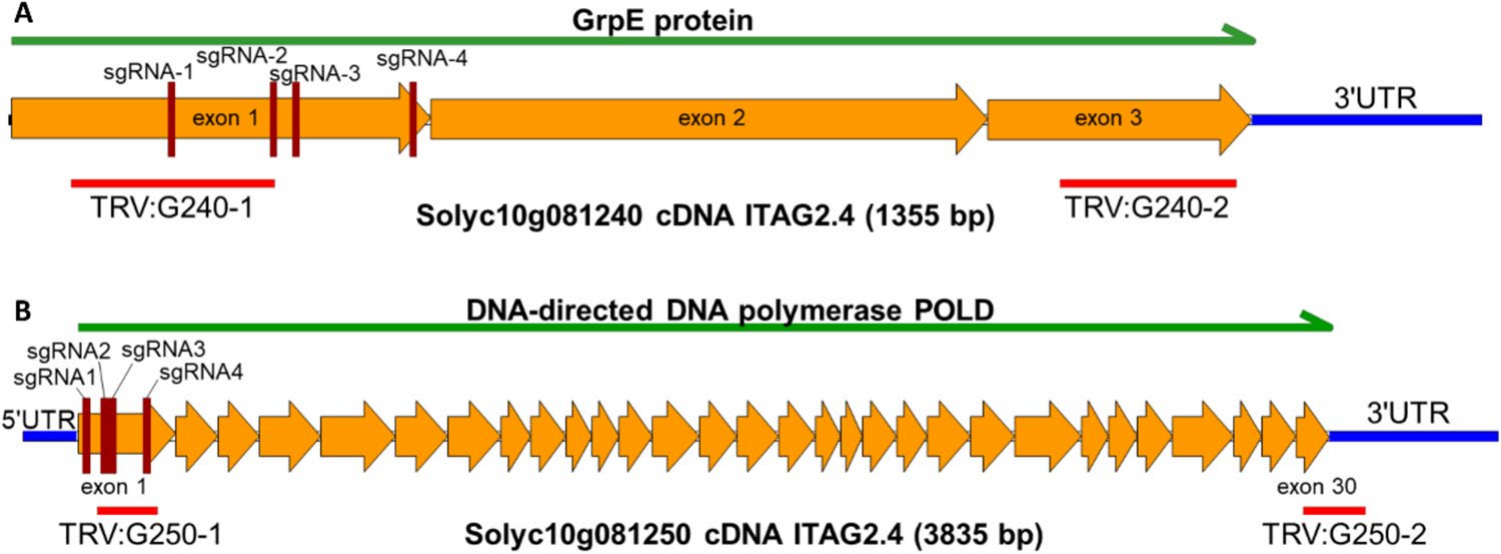
Location of the target regions of the VIGS/RNAi fragments and CRISPR-Cas9 guide RNAs. **A** TRV:G240-1 and TRV:G240-2 represent the VIGS (and RNAi) fragments of Solyc10g081240. **B** TRV:G250-1 and TRV:G250-2 represent the VIGS (and RNAi) fragments of Solyc10g081250. sgRNA1, 2, 3, and 4 represent target sequences of guide RNAs of the CRISPR constructs for both genes

The candidate genes were transiently silenced using a VIGS approach. Two TRV silencing constructs were designed for each of the candidate genes (Fig. 3). In total, VIGS experiments were performed three times on the *Ty-6* lines, with the *Ty-1* line and Moneymaker plants as controls.

In the first VIGS experiment, constructs TRV:G240-2 and TRV:G250-2 were used. Silencing levels were determined at 32 dpi via qRT-PCR (Fig. S5). The expression of Solyc10g081240 and Solyc10g081250 was decreased by 46–89% in TRV:G240 plants and by 19–61% in TRV:G250 plants compared with TRV:GUS plants. This indicates that the silencing constructs effectively reduced the expression of the candidate genes.

**Fig. 4:**
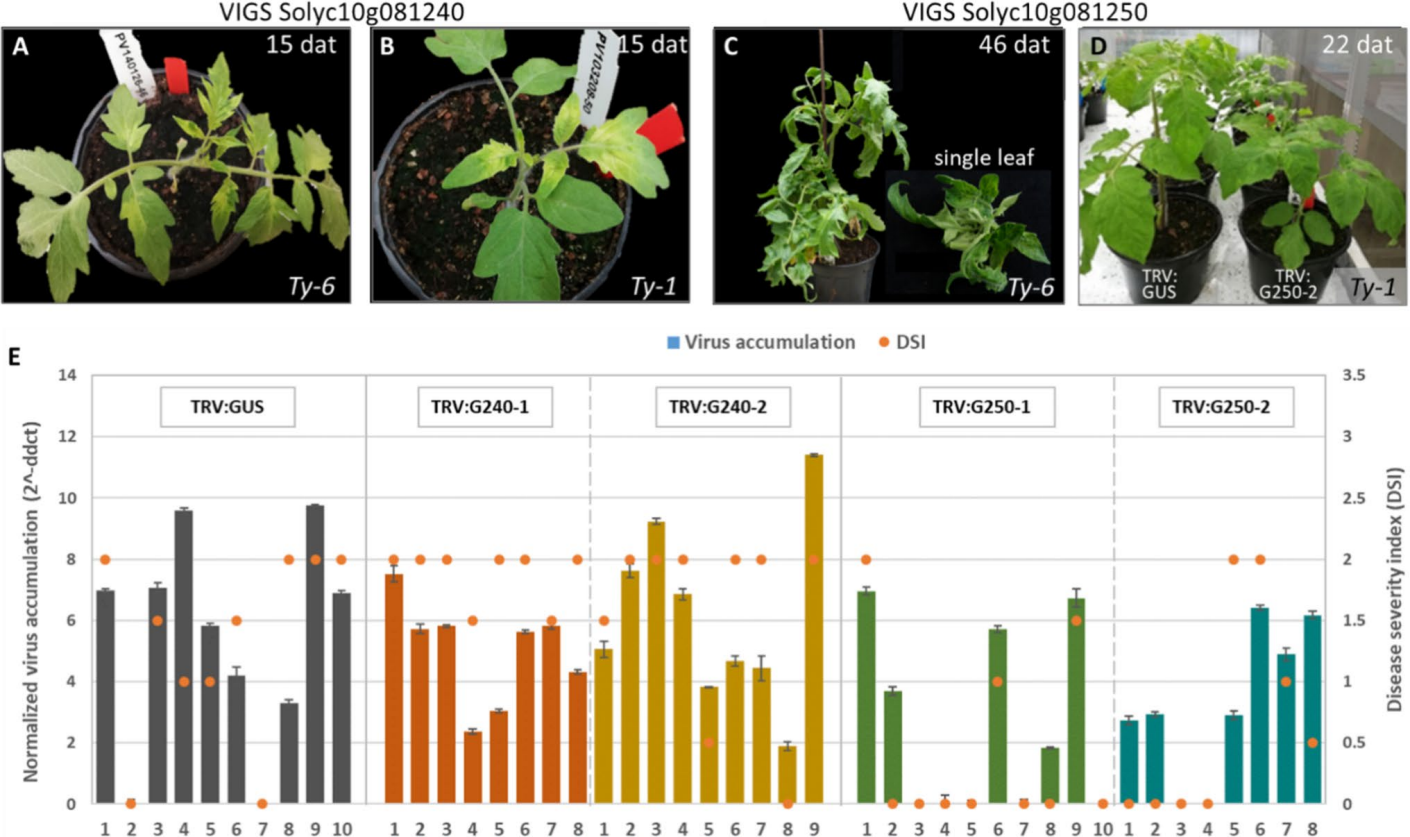
**A-D** Effects of silencing of the candidate genes on plant morphology. All the plants were only inoculated with TRV constructs, not with TYLCV. dat, days after TRV:VIGS inoculation. **A** TRV:G240 infected *Ty-6* plant; **B** TRV:G240 infected *Ty-1* plant; **C** TRV:G250 infected *Ty-6* plants; **D** TRV:G250 infected *Ty-1* plants compared with TRV:GUS control. **E** TYLCV virus accumulation and disease severity index (DSI) in individual TRV-treated and TYLCV-infected *Ty-6* plants at 36 dpi. TRV: GUS plants were included as a control. Bar graphs indicate the relative TYLCV viral DNA accumulation in each plant; scatter spots indicate the DSI scores of each plant

In the second experiment, all four silencing constructs were used. Fig. S6A shows that at least in four out of five plants the target gene showed a reduced expression compared with the GUS control, 12–61% in the TRV:G240 plants and 11–48% in TRV:G250 plants. Silencing Solyc10g081240 caused a clear chlorosis-like effect at an early stage in all genotypes (Fig. 4A&B) which disappeared at 20–30 days after TRV infection. The chlorosis phenotype is consistent with the significant decline in chlorophyll content in heterozygous mutants of Arabidopsis GrpE orthologs (Su et al. 2020). Silencing Solyc10g081250 in *Ty-6* plants resulted in severe stunting and a distorted plant shape (Fig. 4C). Interestingly, silencing Solyc10g081250 in *Ty-1* and MM control plants only led to slightly shorter plants compared to non-silenced plants at the early stage (Fig. 4D) and the difference vanished around 30 days after TRV infection.

To determine whether silencing of the candidate genes would compromise the TYLCV resistance conferred by *Ty-6*, the development of the symptoms was monitored and the level of virus accumulation was determined at 36 dpi (Fig. 4E). No increase in the severity of the TYLCV symptoms was observed in the TRV:G240-treated *Ty-6* plants compared with that in the TRV:GUS control plants. Also, the virus accumulation level in the TRV:G240 plants was comparable with that in control plants. For the TRV:G250-treated *Ty-6* plants, no increased severity of symptoms or higher virus accumulation was found. Unexpectedly, eleven of these plants had a disease severity index (DSI) score of 0 at 36 dpi, of which four plants contained TYLCV and seven plants had no detectable TYLCV accumulation. This was contrasting with the observation that most of the TRV:GUS-treated *Ty-6* plants showed a DSI score of 1–2 with readily detectable viral accumulation. It suggests that silencing Solyc10g081250 in *Ty-6* plants enhanced TYLCV resistance. However, there is also a possibility that the extremely low DSI and lack of viral accumulation in those seven plants were caused by escape from the TYLCV inoculation or sampling errors because of the distorted morphology of the TRV:G250 plants. Reducing the expression level of Solyc10g081240 or Solyc10g081250 did not influence the resistance level in the control *Ty-1* plants nor the TYLCV symptom development in susceptible MM.

In the third VIGS experiment, TRV:G240-2 and TRV:G250-2 were co-infiltrated in *Ty-6* and MM plants. All TRV:G240&G250-treated plants had a chlorotic or/and distorted phenotype. The silencing levels of each candidate gene were analyzed (Fig. S6B). In the TRV:G240&G250 plants #3 and #4, the transcript levels of Solyc10g081250 were reduced by approximately 50%, whereas the transcript levels of Solyc10g081240 were not decreased. The opposite situation was found in TRV:G240&G250 plant #1 and #2. This indicates that in the co-infiltrated plants, TRV:G240-2 and TRV:G250-2 compete for silencing effect.

According to the relative virus accumulation level results at 42 dpi (Fig. S7), four *Ty-6* TRV:GUS plants showed extremely low TYLCV accumulation, and two were TYLCV-free. Three TRV:G240&G250 infected *Ty-6* plants were TYLCV-free. No increased TYLCV symptoms or higher virus accumulation were detected in the TRV:G240&G250-infected *Ty-6* plants compared to TRV:GUS control plants. As indicated in the second VIGS experiment, the distorted morphology may influence TYLCV inoculation efficiency and sampling accuracy.

### Knockdown or knockout transformants of both candidate genes show low regeneration efficiency and viability

In addition to transient silencing, we also tried to obtain stable knock-down/knock-out transformants of the candidate genes in *Ty-6* plants via RNAi and CRISPR-Cas9 approaches. For transformation, RNAi constructs RNAi:G240-2 and RNAi:G250-2, as well as CRISPR-Cas9 constructs CC:G240 and CC:G250 were used (Fig. 3). The regeneration efficiency of all transformed calli was extremely poor (Table S3). Even while the RNAi construct silencing Solyc10g081240 resulted in 37% regeneration efficiency, most calli only produced one leaf instead of full shoots. Light green and white leaves were often observed on the transformants.

After all the effort, only six primary transformants (T1) containing construct RNAi:G240 and two T1 with RNAi:G250 were obtained. Among them only two RNAi:G240 transformants showed reduced expression levels, however, they did not produce T2 seeds. Moreover, for both candidate genes, no mutated plants were regenerated via CRISPR-Cas9-induced mutations.

### Overexpressing the *Ty-6* allele of Solyc10g081250 in MM confers TYLCV resistance

**Fig. 5:**
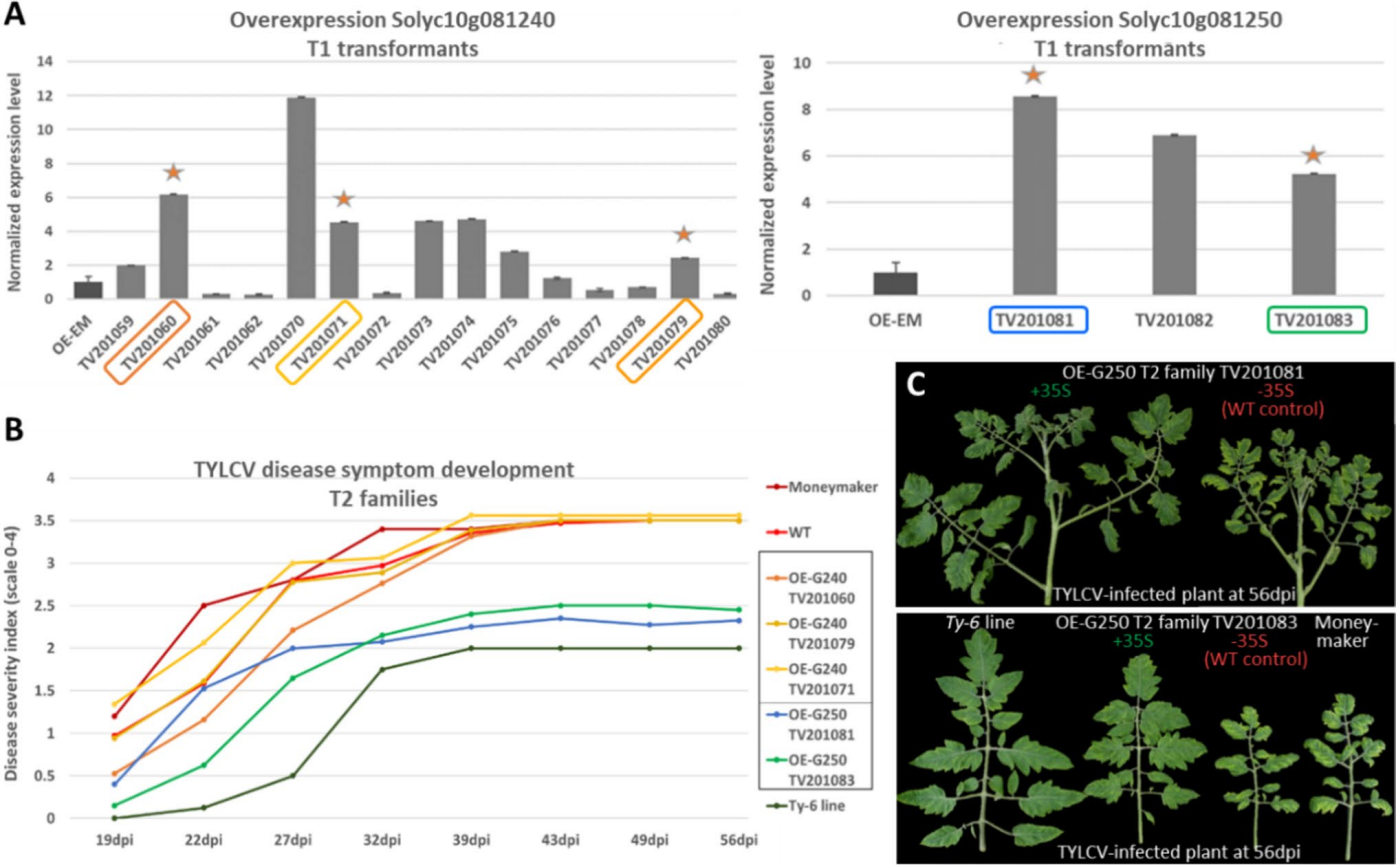
**A** Relative expression levels of *Ty-6* alleles of the candidate genes Solyc10g081240 or Solyc10g081250 in Moneymaker T1 overexpression (OE) transformants. OE-EM control, transgenic plants containing the empty vector. Orange stars indicate T1 transformants with a high relative expression level of which T2 progeny was obtained. TV201070, TV201073, TV201074, TV201075, and TV201082 produced no seeds. **B** Disease severity index (DSI) of transgenic T2 families expressing candidate genes. WT, non-transgenic T2 plants. Compared to WT, the OE-G250 families showed statistically significant reduction in DSI (P < 0.05), except for TV201081 at 22 dpi. From 27 dpi onward, P-values were below 0.0001. In contrast, the OE-G240 families showed no significant reduction in DSI. **C.** TYLCV symptom expression of transgenic T2 plants from families TV201081 and TV201083 overexpressing the *Ty-6* allele of Solyc10g081250 in MM background compared with the non-transgenic control T2 plants (without 35S promotor [-35S]) at 56 days post TYLCV inoculation

As shown above, it seemed impossible to obtain transgenic plants showing silencing or mutation of either of the two candidate genes due to their crucial roles in plant development. Therefore, we overexpressed the *Ty-6* alleles of the two genes in TYLCV-susceptible MM backgrounds. T1 transformants that had higher expression levels of Solyc10g081240 (OE-G240) or Solyc10g081250 (OE-G250) than that of control were selected (Fig. 5A) to produce T2 transgenic families.

TYLCV disease assays were performed on the selected T2 families (labeled with stars in Fig. 5A); three OE-G240 T2 families (TV201060, TV201071, and TV201079) and two OE-G250 T2 families (TV201081 and TV201083). As T2 progeny segregate for the presence of the T-DNA insertion(s), a PCR was performed using 35S promotor primers. The non-transgenic T2 plants (− 35S) were included as an additional negative control, which was referred to as wild-type (WT). There was no significant difference in disease severity between the OE-G240 T2 plants and the WT control (Fig. 5B). At 56 dpi, the average DSI of all three OE-G240 T2 families reached around 3.5, which is the same as the WT control and MM.

In contrast, in the case of OE-G250 families, at 19 and 22 dpi, TV201083 showed less severe TYLCV symptoms than the WT controls and the OE-G240 T2 plants (Fig. 5B). From 27 dpi onward, the disease severity of the OE-G250 families was significantly lower than that of the WT controls (P values < 0.0001) and MM, while slightly (though statistically significant) higher than the DSI scores (≤ 2) of the *Ty-6* line, except at 32 dpi. The leaves of the infected MM plant showed clear symptoms including leaf yellowing, curling, and reduction in leaf size (Fig. 5C, lower right). In contrast, the leaves of the infected OE-G250 T2 plants (+ 35S) displayed only some yellowing, which was less severe compared with the WT controls and MM.

**Fig. 6:**
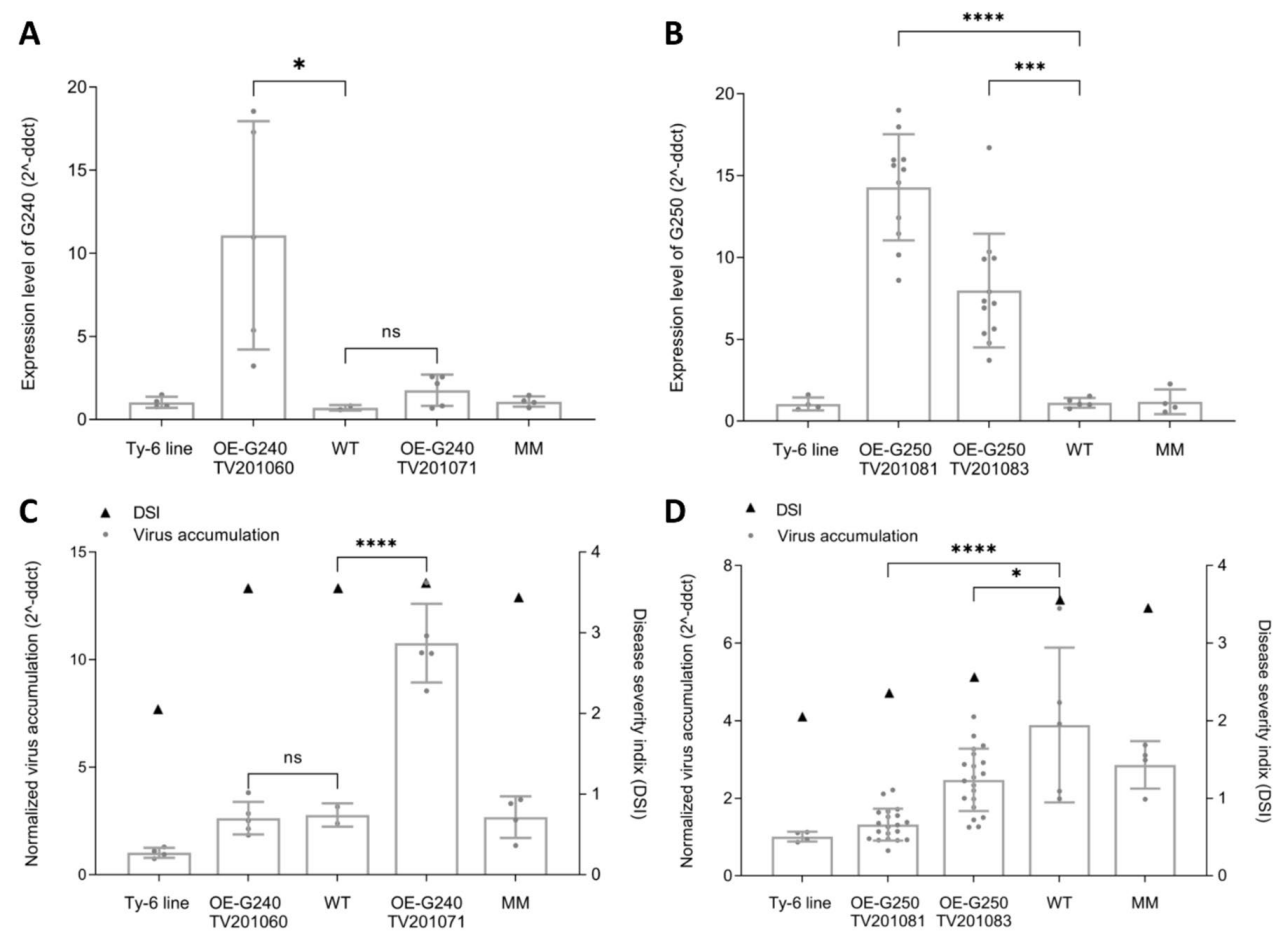
Expression levels of *Ty-6* candidate genes and TYLCV virus accumulation in the transgenic T2 plants. The bar graphs in **A** and **B** indicate the relative expression level of Solyc10g081240 and Solyc10g081250 respectively. Bars in **C** and **D** show the virus accumulation level, while the triangles indicate the disease severity index (DSI) at 56 dpi. All the values were normalized relative to the *Ty-6* plants. Statistical differences were calculated for gene expression level **A**,**B** and virus accumulation **C**,**D**; * = *P* < 0.05, *** = *P* < 0.001, **** = *P* < 0.0001, ns = not significant. OE-G240 and OE-G250, transgenic T2 plants overexpressing the *Ty-6* allele of Solyc10g081240 or Solyc10g081250 in MM background, respectively; WT, non-transgenic T2 plants; MM, Moneymaker

In the two OE-G240 T2 families, a significantly higher expression of Solyc10g081240 was found only for TV201060 compared to the WT control (Fig. 6A). On the other hand, viral accumulation levels of TV201060 and the WT control were comparable to that in MM but higher than that in the *Ty-6* line (Fig. 6C). Intriguingly, the expression of Solyc10g081240 in TV201071 was not significantly changed, however, the virus accumulation was higher compared to controls. In the OE-G250 transgenic plants, the expression levels of Solyc10g081250 were highly elevated compared with the WT control (Fig. 6B). Correspondingly, significantly lower viral accumulation was detected in these plants (Fig. 6D). Moreover, the viral accumulation appears to be negatively associated with the expression level of (the *Ty-6* allele of) Solyc10g081250.

The TYLCV disease assay was repeated on the same T2 families. All OE-G240 plants displayed TYLCV symptoms similar to non-transgenic plants (Fig. S8A), while the OE-G250 transgenic T2 plants showed clear segregation of symptoms (Fig. S8B). It is unclear whether this segregation pattern correlates with a homozygous/ heterozygous T-DNA insertion or with the expression level.

### Homozygous MM transgenic plants overexpressing Solyc10g081250 from *Ty-6* display comparable TYLCV resistance levels as *Ty-6* plants

From the first TYLCV disease assay on OE-G250 T2 families (TV201081 and TV201083), six plants from each family were kept to generate T3 families (Table S4). Among them, T2 plants TV201081-18, TV201081-19, and TV201083-9, with relatively high expression of Solyc10g081250, showed similar levels of virus accumulation as *Ty-6* plants (0.65–1.25). Therefore, the T3 families TV212061, TV212060, and TV212057 derived from these T2 plants were used to evaluate the resistance levels against TYLCV, with non-transgenic families TV212059 and TV212058 as wild-type controls. Given that all germinated T3 plants (24 to 54 plants per family, Table S5) were positive for 35S PCR amplification, the tested OE-G250 T3 families and their T2 parents are most likely homozygous for the Solyc10g081250 insertion. Taken together with the low viral accumulation and DSI in the three T2 plants, homozygous OE-G250 transgenic plants appeared to be more resistant to TYLCV than hemizygous OE-G250 transgenic plants, and their resistance levels were comparable to *Ty-6* plants.

**Fig. 7:**
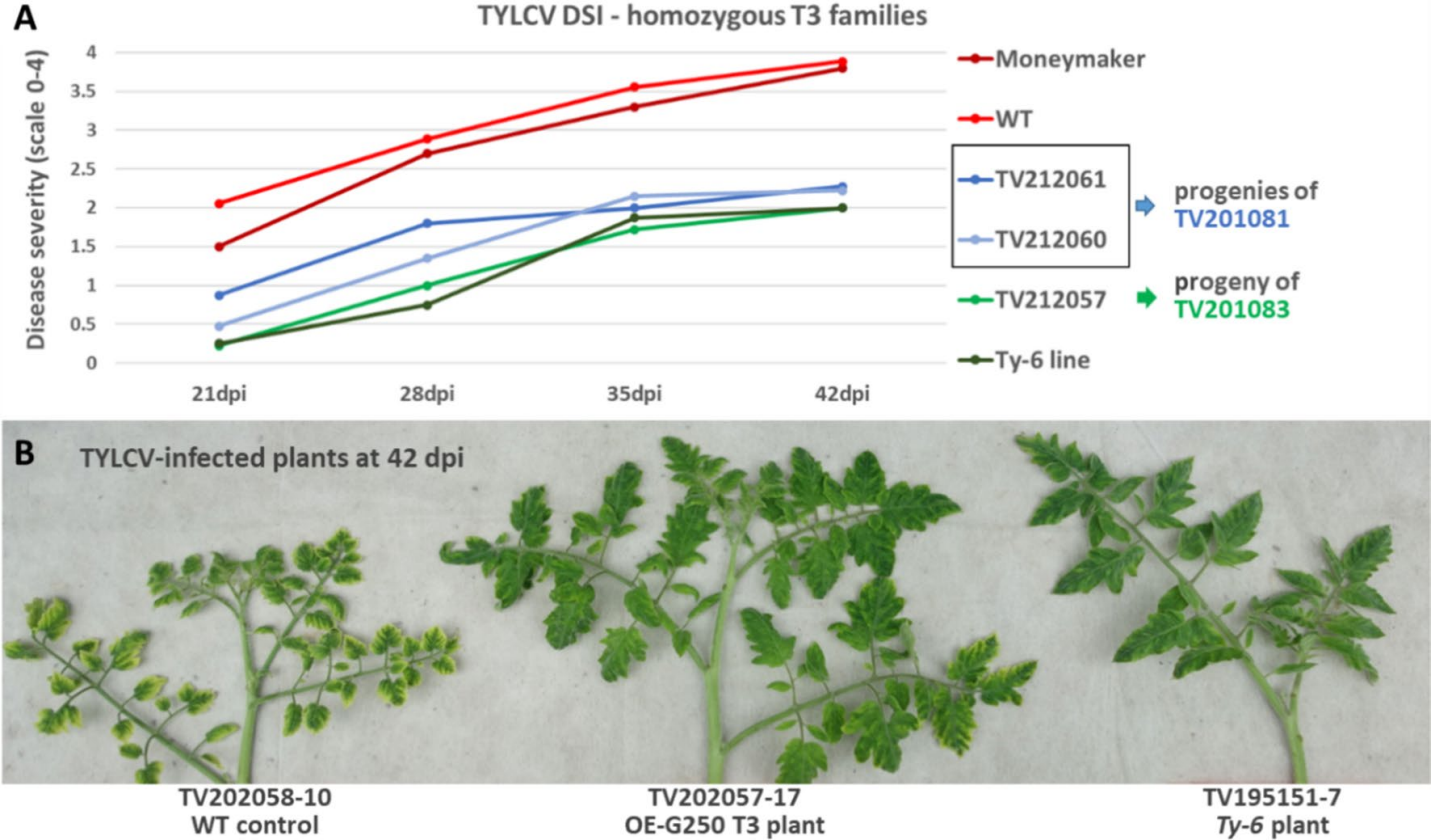
TYLCV symptoms and symptom development in homozygous T3 families. **A.** the symptom development in homozygous T3 families. The DSI of the homozygous T3 families exhibited a statistically significant reduction compared to WT, with P < 0.0001 at all time points. Compared to *Ty-6* plants, T3 families showed no significant difference in DSI, except for TV212061 at 21 dpi (*P* = 0.02) and 28 dpi (*P* = 0.03). **B** TYLCV symptom expression in a homozygous T3 plant compared to the wild-type control and *Ty-6* plant at 42 dpi

To validate this finding, the development of the TYLCV symptoms in the homozygous transgenic T3 families was monitored and compared with that in the *Ty-6* plants. TV212057 (green line in Fig. 7A) was derived from TV201083 (green line in Fig. 5B); T3 families TV212060 (blue line in Fig. 7A) and TV212061 (light blue line in Fig. 7A) were derived from TV201081 (blue line in Fig. 5B). The line graphs reveal that while the segregating T2 family TV201083 (20 plants) showed higher average DSI scores than *Ty-6* plants over the time points (green line in Fig. 5B), symptom development in the homozygous T3 family TV212057 (green line in Fig. 7A) was almost identical to that in the *Ty-6* plants. Meanwhile, T3 family TV212060 showed a similar trend as *Ty-6* plants in the development of symptoms with slightly higher DSI averages. The average symptom severity of TV212061 T3 plants reached 2 at around 28 dpi, which was about 7 days earlier than other T3 families and *Ty-6* plants, but remained around 2 for the remaining period. On the other hand, the average DSI of segregating T2 family TV201081 (20 plants) rose to 2 approximately 12 days earlier than the *Ty-6* plants and stayed higher than the *Ty-6* plants over the following time points (blue line in Fig. 5B).

Overall, the DSI of the T3 families was significantly different from that of the WT at all time points, while there was no significant difference in DSI between T3 families and the *Ty-6* line, except for TV212061 at 21 and 28 dpi.

As indicated above, the development of symptoms in the homozygous transgenic T3 families is more comparable with *Ty-6* plants than in the segregating T2 families, which might be due to the presence of hemizygous plants in the T2 families. Moreover, the segregation of the symptoms in the OE-G250 transgenic T2 plants was not observed in homozygous T3 families. Instead, all the T3 plants showed similar expression of symptoms as TYLCV-infected *Ty-6* plants (Fig. 7B).

### *Ty-6* gene provides broad-spectrum resistance

**Fig. 8:**
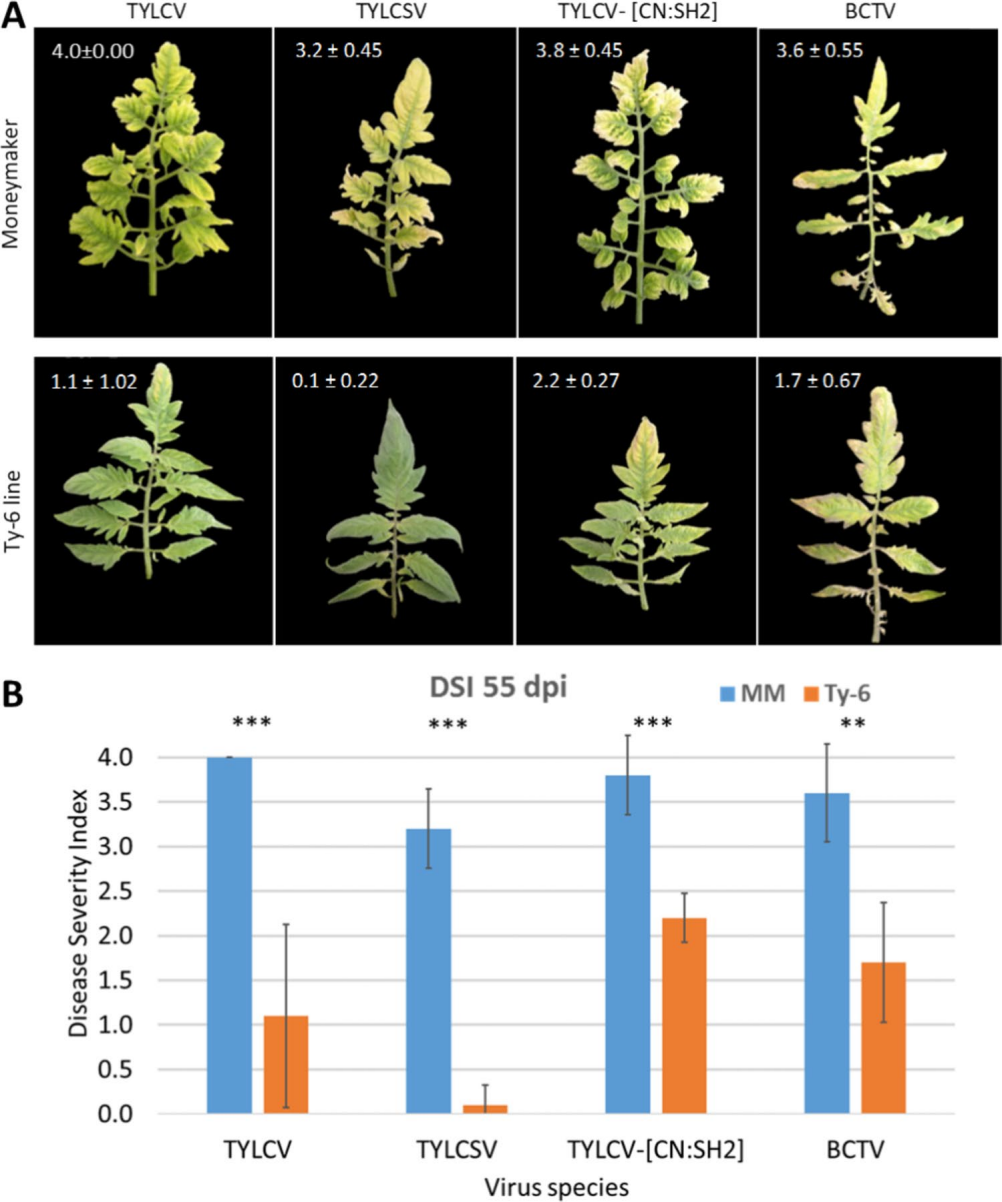
**A** The resistance response of the *Ty-6* line to *Begomovirus* TYLCV-Israel strain (TYLCV-IL), tomato yellow leaf curl Sardinia virus (TYLCSV), tomato yellow leaf curl virus—China: Shanghai2 isolate (TYLCV- [CN:SH2]) and *Curtovirus* beet curly top virus (BCTV) at 55 dpi. The photo of Moneymaker infected with TYLCV was adopted from Shen et al. (2020). **B** The disease severity represents the mean of five individual plants ± standard deviation. Asterisks indicate significant difference between the MM and *Ty-6* genotypes for each tested virus according to one-way ANOVA. The number of asterisks reflects the level of significance (** = *p* < 0.01; *** = *p* < 0.001)

To evaluate the resistance spectrum of the *Ty-6* gene, we tested the *Ty-6* line with different strains of *Begomovirus* including TYLCV-Israel strain (TYLCV-IL), Tomato Yellow Leaf Curl Sardinia virus (TYLCSV), TYLCV-China: Shanghai2 isolate (TYLCV-[CN:SH2]) and *Curtovirus* beet curly top virus (BCTV). As a control, MM was included which showed full susceptibility to all tested viruses (mean DSI = 3.2–4.0). Compared to MM, the *Ty-6* line displayed fewer symptoms with the TYLCV and TYLCSV strains and after BCTV infection (Fig. 8). Furthermore, the TYLCV accumulation was quantified in systemic leaves (Fig. S9). On average, the viral accumulation in *Ty-6* plants was around 70% lower than that in MM.

As the *Ty-6* gene provides broad-spectrum resistance, including to TYLCSV, we expected that overexpression of the *Ty-6* allele of Solyc10g081250 will also confer resistance to TYLCSV. Thus, we performed TYLCSV disease assays on T3 families with non-transgenic plants as wild-type control. As expected, MM transgenic plants overexpressing Solyc10g081250 were highly resistant to TYLCSV (Fig. S10). This gives weight to our conclusion that Solyc10g081250 is the resistance gene in *Ty-6* plants.

### New in-gene CAPS marker for *Ty-6*

As *Ty-6* is an interesting gene conferring broad-spectrum resistance against geminiviruses we developed an in-gene CAPS marker based on the SNP in exon 13, to be used on gDNA. Primers were developed in introns 12 and 14 (Table S2, Fig. S11). After digestion of the 609-bp PCR product with restriction enzyme *Psi*I two fragments of 370 bp and 239 bp are obtained with MM gDNA, while the PCR product of *Ty-6* remains undigested.

### The Y515H variant in the *Ty-6* allele may impact the function of POLD1 in DNA replication

Solyc10g081250 encodes the tomato homolog of the DNA polymerase delta (Polδ) subunit 1 (*SlPOLD1*) with 84% identity to Arabidopsis POLD1, AT5G63960, also known as EMB2780. We identified two conserved functional domains in tomato POLD1, catalytic domain (interval 567–960) and exonuclease domain (interval 290–518) (Fig. S12). The exonuclease domain has a proofreading (3’-5’ exonuclease) ability, which is important for the fidelity of DNA replication (Bębenek and Ziuzia-Graczyk 2018). The amino acid showing variation in *Ty-6*, Y515H, is located at the edge of the exonuclease domain of tomato POLD1 (Fig. S12). Therefore, the variant might impact the proofreading activity and decrease the fidelity of TYLCV DNA replication.

### Tomato *POLD1* allelic variants in wild accessions

To explore natural tomato *POLD1* genetic variants and discover novel *POLD1* alleles against TYLCV, we performed allele mining. In the coding regions, 38 non-synonymous variants (Fig. S11) were identified in 35 wild accessions compared to the Heinz 1706 reference genome (Table S6). Importantly, no Y515H variant was found among these natural variants. The Y515H variant was also absent from *S. chilense* accessions LA1938 and LA2779, in the pedigree of the *Ty-6* lines from Florida (Fig. S1A), indicating that *Ty-6* was introduced from ‘Tyking’ and from an Israeli breeding line of undetermined pedigree.

Three variants (V523I, K635N, and G751S) may lead to protein dysfunction according to the two SNP effect prediction programs. Particularly, V523I was predicted to be deleterious with a high probability, of which the PolyPhen-2 score is extremely close to that of Y515H. Additionally, V523I, which is close to Y151H, was only discovered in *S. lycopersicum* LA1479. Amino acid variants K635N and G751S in the catalytic domain appear to be species-specific, as they are present in all sequenced *S. chmielewskii* (two accessions: LA2663 and LA2695) and *S. habrochaites* (seven accessions), respectively. Besides, eight variants (indicated in Table S6) were predicted to impact protein function by Panther.

## Discussion

### POLD1 plays a critical role in plant development

In eukaryotes, Polα, Polδ, and Polɛ, as members of the DNA polymerase B family, are thought to be in charge of genome replication (Pedroza-Garcia et al. 2019). In Arabidopsis and rice, the core DNA replication genes were identified and characterized, including the subunits of Polα, Polδ, and Polɛ, which mediate the elongation of DNA replication (Shultz et al. 2007). In this study, we identified Solyc10g081250, the homolog of the catalytic subunit of Polδ (POLD1) in tomato, as the gene underlying *Ty-6*.

Similar to human and yeast POLD1, tomato POLD1 is predicted to contain two functional domains: a catalytic domain (DNA polymerase activity) and an exonuclease domain (3′ → 5′ exonuclease activity). In eukaryotes, via DNA polymerase activity, Polδ synthesizes Okazaki fragments on the lagging strand (Burgers et al. 2009) and participates in the initiation and termination of the leading strand (Garbacz et al. 2018; Zhou et al. 2019). Meanwhile, with 3′ → 5′exonuclease activity, it proofreads errors made by Polɛ and Polα (Bębenek and Ziuzia‑Graczyk 2018; Pavlov et al. 2006). Besides, Polδ displaces the RNA/DNA primers in the process of Okazaki fragment maturation (Maga et al. 2001), followed by the degradation of the displaced primers and ligation of DNA-DNA nick (Burgers and Kunkel 2017; Giannattasio and Branzei 2019; Prindle and Loeb 2012). Next to its function in DNA replication, Polδ also plays a role in DNA repair and genome stability (Pedroza-Garcia et al. 2019; Prindle and Loeb 2012).

To study the role of POLD1 in TYLCV infection, we endeavored but were unable to produce *slpold1* knockdown/out transgenic plants. Meanwhile, *Ty-6* plants with transient silencing of *POLD1* exhibited severe stunting and a distorted phenotype. These results are consistent with the earlier discovery that homozygous *pold1* mutants could not be generated in Arabidopsis and the reduction of *POLD1* expression caused higher genome instability in plants with weak *pold1* alleles (Schuermann et al. 2009). We hypothesize that *Ty-6* plants similarly contain a weak *pold1* allele compared to MM and *Ty-1* plants. This would explain why reduction of *POLD1* expression in *Ty-6* background caused a much more severe phenotype than in MM and *Ty-1* background, as shown in Fig. 4C and D.

So far, gigantea suppressor 5 (*gis5*) is the only viable hypomorphic allele (A707V) of *POLD1* in Arabidopsis. The A707V mutant showed early flowering and leaf curling phenotypes at 24 °C and arrested development at 28 °C (Iglesias et al. 2015). In our study, we identified a homozygous variant (Y515H) of *POLD1* in tomato, which caused no pleiotropic effects on plant development. The possible explanation is that unlike A707V located in the conserved catalytic polymerase domain, the Y515H variant in the *Ty-6* allele was not one of the most highly conserved amino acid residues in the exonuclease domain.

In addition to its key role in DNA replication, POLD1 may also function in the establishment of transcriptionally active epigenetic marks during DNA replication, which affects some specific loci and gene expression, and further affects plant development (Iglesias et al. 2015). Given that the *Ty-6* allele of *POLD1* has no effect on plant growth, it is most likely true that the Y515H variation has no impact on the POLD1 function in the deposition of epigenetic marks.

### Viruses hijack host POLD1 for viral DNA replication

With a small genome size, geminiviruses only encode a limited number of proteins. The multifunctional roles of geminivirus‑encoded proteins compensate for the limited coding capacity (Devendran et al. 2022). Only two viral factors have been identified to play roles in viral DNA replication: the replication initiator protein (Rep/C1) (Ruhel and Chakraborty 2019) and replication enhancer protein (REn/C3) (Settlage et al. 2005). However, neither of them has DNA polymerase activity. Therefore, DNA replication of geminiviruses relies on the host genome replication machinery (Gutierrez 1999).

To understand the geminivirus replication mechanism, Li et al. (2019) identified 131 yeast genes probably involved in geminivirus replication using the yeast *ts* mutant library. Among these were POL3 (*Saccharomyces cerevisiae* POLD1) and POL1 (*S. cerevisiae* POLA1). In this study, we found that virus accumulation in *Ty-6* plants was significantly lower than that in MM, which suggests that the *Ty-6* allele of POLD1 (a weak *pold1* allele) impairs viral DNA replication. Meanwhile, the MM allele of POLD1 is probably involved in the viral replication. This is supported by our VIGS result that silencing Solyc10g081250 in *Ty-6* plants enhanced TYLCV resistance rather than reduced it. Since *Ty-6* only confers moderate resistance, most likely the *Ty-6* allele still allows a certain partial function required for TYLCV replication, and knocking it down results in the loss of the remaining function. In this case, we would expect that silencing of Solyc10g081250 in MM results in lower susceptibility, however the TYLCV symptoms of these plants did not decrease compared to wild-type plants. Meanwhile, the MM plants infected with the VIGS construct targeting Solyc10g081250 only showed slight stunting, as mentioned above. A possible explanation is that the expression of the MM allele was not sufficiently suppressed to undermine viral replication.

Notably, homozygous transgenic MM plants overexpressing the *Ty-6* allele of *POLD1* showed a comparable level of resistance as *Ty-6* plants (Fig. 7). This seems to be conflicting with the observation that expression of *POLD1* gene Solyc10g081250 significantly increased at 28 days after TYLCV inoculation compared with mock treatment in MM, while expression in the *Ty-6* line was not increased (Fig. S4B). However, in the homozygous MM OE-G250 transgenic plants the POLD1 protein encoded by the *Ty-6* allele outcompetes the protein encoded by the endogenous MM allele. When we consider that the *Ty-6 pold1* allele encodes a protein that is less functional with regard to viral DNA replication, this would explain why the MM OE-G250 transformants show similar resistance to TYLCV as the *Ty-6* line.

Our findings are in agreement with a recent report by Wu et al. (2021). They found that knocking down *POLD1* and *POLD2* by VIGS in *N. benthamiana* reduces the local accumulation of geminivirus. As known, viral DNA replication mainly occurs via two steps: conversion of ssDNA to a dsDNA intermediate via complementary strand synthesis, and synthesis of ssDNA from a dsDNA intermediate through a rolling-circle mechanism (RCR) (Gutierrez 1999, 2004; Hanley-Bowdoin et al. 2010). Via two-step anchored qPCR in POLD2-silenced plants, Wu et al. (2021) found that DNA polymerase δ does not interfere with the complementary strand synthesis, but only assists the synthesis of new viral ssDNA in RCR. This could also explain our observation that the *Ty-6* plants still allow a certain amount of virus accumulation. In fact, RCR is not the only mode of geminivirus replication. Jeske et al. (2001) revealed the involvement of recombination-dependent replication (RDR) as well, but with a minor role in geminivirus replication (Kaliappan et al. 2012). Polδ performs recombination-associated DNA synthesis to rescue arrested replication forks (Naiman et al. 2021). Therefore, Polδ could also be hijacked by geminivirus as the DNA polymerase in RDR replication. If so the *Ty-6* allele of POLD1 might impair geminivirus replication in both RCR and RDR modes.

In addition to the plant host, TYLCV may also rely on the Polδ-mediated replication machinery in the whitefly vector. As recently reported (He et al. 2020), TYLCV promotes the expression of the whitefly Polδ subunits 2 and 3. On the other hand, knocking down the subunits resulted in a decreased TYLCV load. Of note, knocking down the subunits does not affect the viral load of geminivirus papaya leaf curl China virus (PalCuCNV) (He et al. 2020). Until now, the potential geminivirus replication in whiteflies has only been indicated in TYLCV family members (Czosnek et al. 2021; Pakkianathan et al. 2015; Sinisterra et al. 2005; Wang et al. 2016). The broad resistance spectrum of *Ty-6* (TYLCV, TYLCSV, TYLCV- [CN:SH2] and BCTV), and the requirement of Polδ in viral replication of Tomato golden mosaic virus (TGMV) (Wu et al. 2021) and Cassava Mosaic Geminiviruses (CMG) (Lim et al. 2022), however, suggest that geminiviruses replication in plants seems to occur by a conserved host Polδ-dependent replication system.

It is very interesting that *Ty-6* hinders viral DNA replication of different types of geminivirus at varying levels, i.e. TYLCV-IL vs. TYLCSV. The exact mechanism by which *Ty-6* can interact differently with distinct geminiviruses is unknown. One possible way is via the interaction between viral proteins and host cellular factors. For instance, both Rep and REn could interact with PCNA (Castillo et al. 2003; Settlage et al. 2005), which is required for the catalytic activity of Polδ as a processivity factor (Mondol et al. 2019; Stodola and Burgers 2016). Rep protein can specifically reduce the sumoylation of PCNA to modulate its function (Arroyo-Mateos et al. 2018). Moreover, REn protein is found to recruit Polδ over nonproductive Polɛ during geminivirus replication (Wu et al. 2021). Therefore, the Rep or Ren differences among distinct geminiviruses may influence the replication efficiency of Polδ, especially the 134–183 amino acid of Rep (Bagewadi et al. 2004) and the hydrophobic residues in the middle of REn (Settlage et al. 2005) that is known to interact with PCNA.

As predicted, the Y515H variant is located in the exonuclease domain of tomato POLD1. In yeast, POL3 mutants in exonuclease active sites elevated mutation rates (Morrison et al. 1993; Nick McElhinny et al. 2007). Theoretically, the Y515H variant may introduce more replication errors in both plant and virus replication because of the impact on the proofreading activity. Surprisingly, it specifically hinders viral genome replication.

In eukaryotes, Tyrosine, Valine, and Isoleucine (amino acids with hydrophobic side chains) are common amino acids present at the position comparable to tomato 515 in POLD1 protein. However, Histidine (an amino acid with an electrically charged side chain) was predicted to be harmful to POLD1. It is possible that Y515H might influence the stabilization of protein structure and the specificity of the interaction of proteins with other biomolecules.

Recently, Lim et al. (2022) identified six mutations in the cassava ortholog of POLD1 that co-segregate with Cassava Mosaic Geminiviruses (CMG) resistance, including V528L (Tomato: V523), L598W (Tomato: L593), G680V/R (Tomato: G675), A684G (Tomato: A679; Arabidopsis: A707V [Iglesias et al. 2015]), and L685F (Tomato: L680). Interestingly, the V523I variant of tomato POLD1 in wild tomato accession LA1479 is corresponding to the V528L mutant in Cassava. Taken together with the close localization to Y515H and the predicted high impact on POLD1 function, V523I is another likely resistant *POLD1* variant. If this variant can be confirmed to be present in LA1470 plants it will be interesting to test those for geminivirus resistance.

Because of the essential role of DNA polymerases in plant development, homozygous mutants without pleiotropic effects have never before been generated or identified. Plants homozygous for the POLD1 variant Y515H, and possibly V532I, identified in this study are good plant models to explore the replication mechanism of geminiviruses in tomato and figure out the novel resistance mechanism underlying POLD1 TYLCV-resistant alleles. Compared to roughly eliminating the function of the target gene, reducing or partly modifying the gene function via single-nucleotide substitutions might be the better way for gene functional studies.

### Use of *Ty-6* in breeding

We identified a variant of *POLD1* as the gene controlling *Ty-6*-mediated resistance in tomato. Interestingly, pedigree results suggest that both *Ty-6* and *ty-5* (Lapidot et al. 2015) are derived from cultivar ‘Tyking’, and both have SNPs that cannot be traced back to any landrace or wild tomato accession. This suggests that these SNPs may have been newly generated in the ancestry of ‘Tyking’.

Different from *ty-5* (*pelota*) involved in the translation machinery, the *Ty-6* allele of POLD1 (partially) inhibits viral replication in the plant host, which reveals a novel antiviral mechanism. *Ty-6* displays broad and durable resistance, but no deleterious effects on plant morphology. Additionally, as an incompletely dominant gene with an additive effect when combined with other resistance genes, it has an advantage over recessive resistance genes in the utilization of breeding for virus resistance. According to the different replication systems of RNA and DNA viruses, *Ty-6* may not confer resistance to RNA viruses. Similarly, it is unknown whether *Ty-6* would be functional against dsDNA viruses. The allele mining on 150 (wild) tomato accessions revealed new *POLD1* alleles that might confer resistance to geminiviruses, which widen the resistance source for breeding.

Meanwhile, more mutants of other polymerases involved in the replication system can be identified using the sequencing databases or created via gene editing, for example, Polα, which is involved in the complementary strand synthesis of viral DNA. Additionally, recently Siskos et al. (2023) identified another host gene required for geminiviral replication, the DNA primase large subunit *PriL*. A variant of this gene in melon conferred recessive resistance against ToLCNDV. Possibly, combining resistance genes that are involved in two different stages of viral replication in the breeding program might result in a high-level resistance against geminiviruses.

## Supplementary Information

Below is the link to the electronic supplementary material.

Supplementary file1 (DOCX 7958 kb)

Supplementary file2 (PDF 179 kb)

## Data Availability

The datasets generated during and/or analysed during the current study are available from the corresponding author on reasonable request.
