# Supplementary material for: The tomato gene Ty-6, encoding DNA polymerase delta subunit 1, confers broad resistance to Geminiviruses
Source: Theor Appl Genet. 2025 Jan 8;138(1):22. doi: 10.1007/s00122-024-04803-w (PMC11711579; doi:10.1007/s00122-024-04803-w)
Supplement: Supplementary file 1 — Supplementary file1 (DOCX 7958 kb) [file 122_2024_4803_MOESM1_ESM.docx]

**Supplementary Figures**


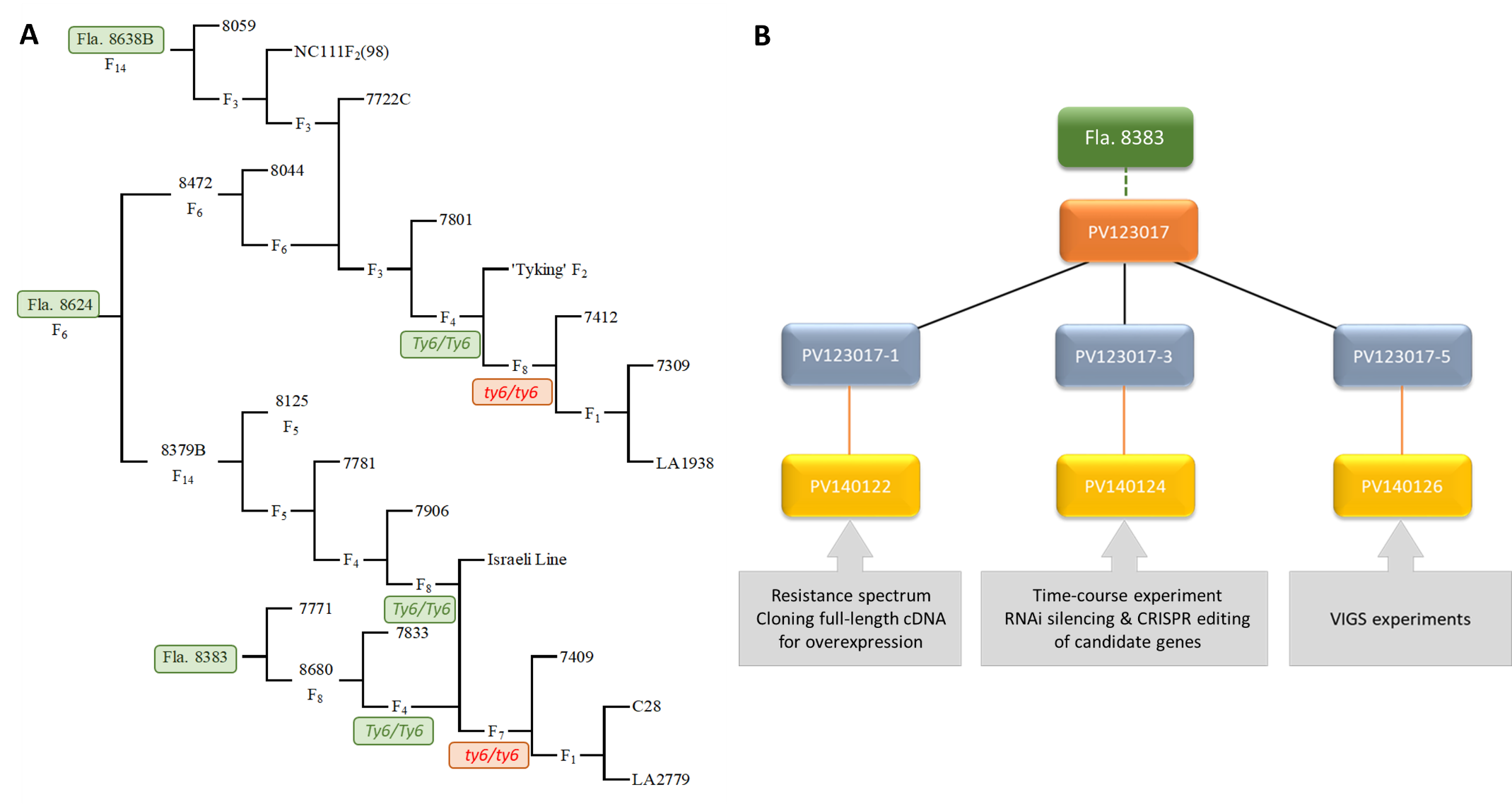


**Fig. S1** **A.** Pedigrees of Fla. 8638B, Fla. 8624 and Fla. 8383, each indicating the point(s) at which *Ty-6* was introduced from ‘Tyking’ or from an Israeli breeding line of undetermined pedigree. **B.** The *Ty-6* homozygous introgression lines used in different experiments in Wageningen.


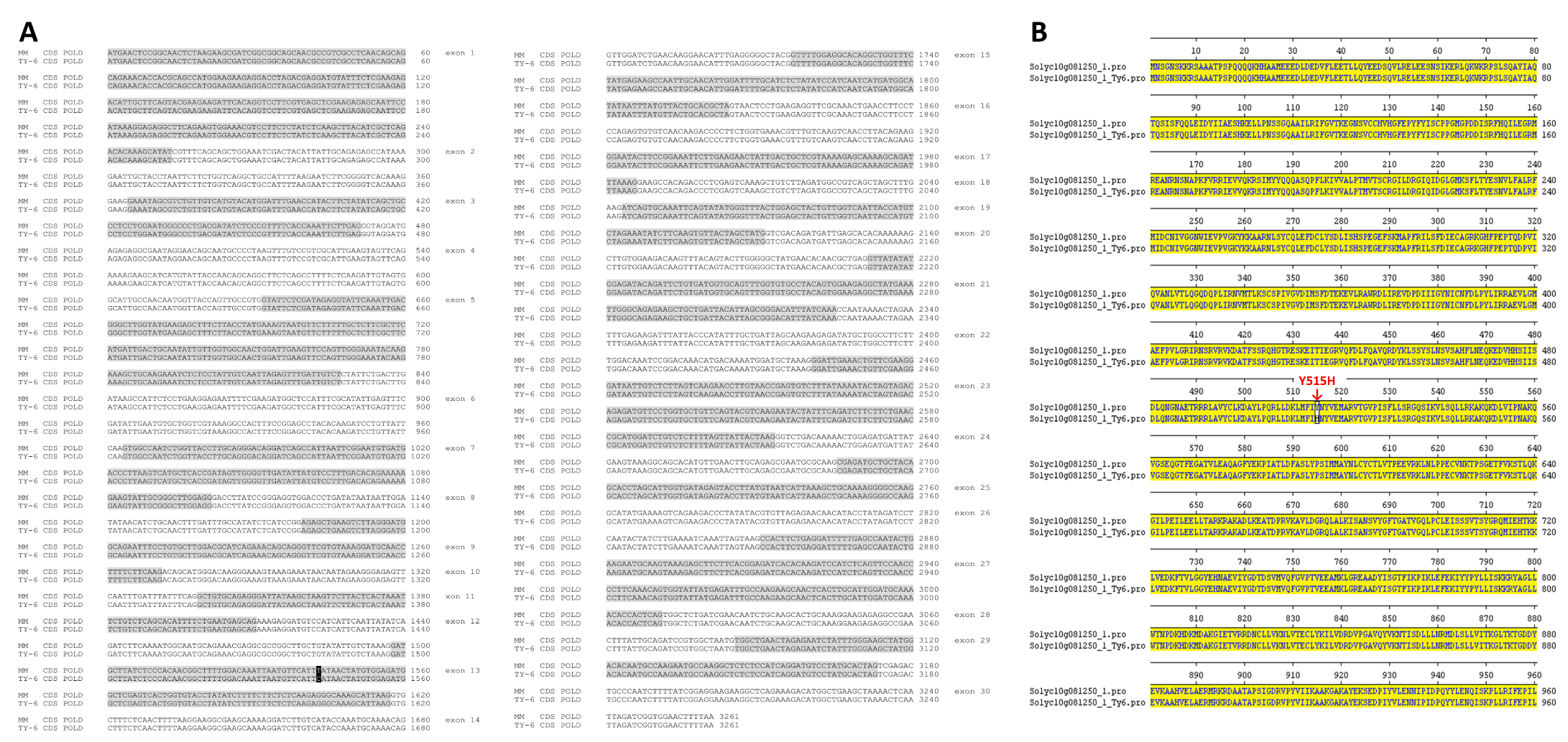


**Fig. S2** **A.** Alignment of the Solyc10g081250 (*POLD*) coding sequences from susceptible breeding line Moneymaker (MM) (upper sequence) and the *Ty-6* line (lower sequence). Different exons are indicated. The T→C SNP in exon 13 is highlighted. **B.** Alignment of the Solyc10g081250 protein sequences from Heinz and susceptible breeding lines (upper sequence) and the *Ty-6* line (lower sequence). Amino acid change Y515H is indicated.


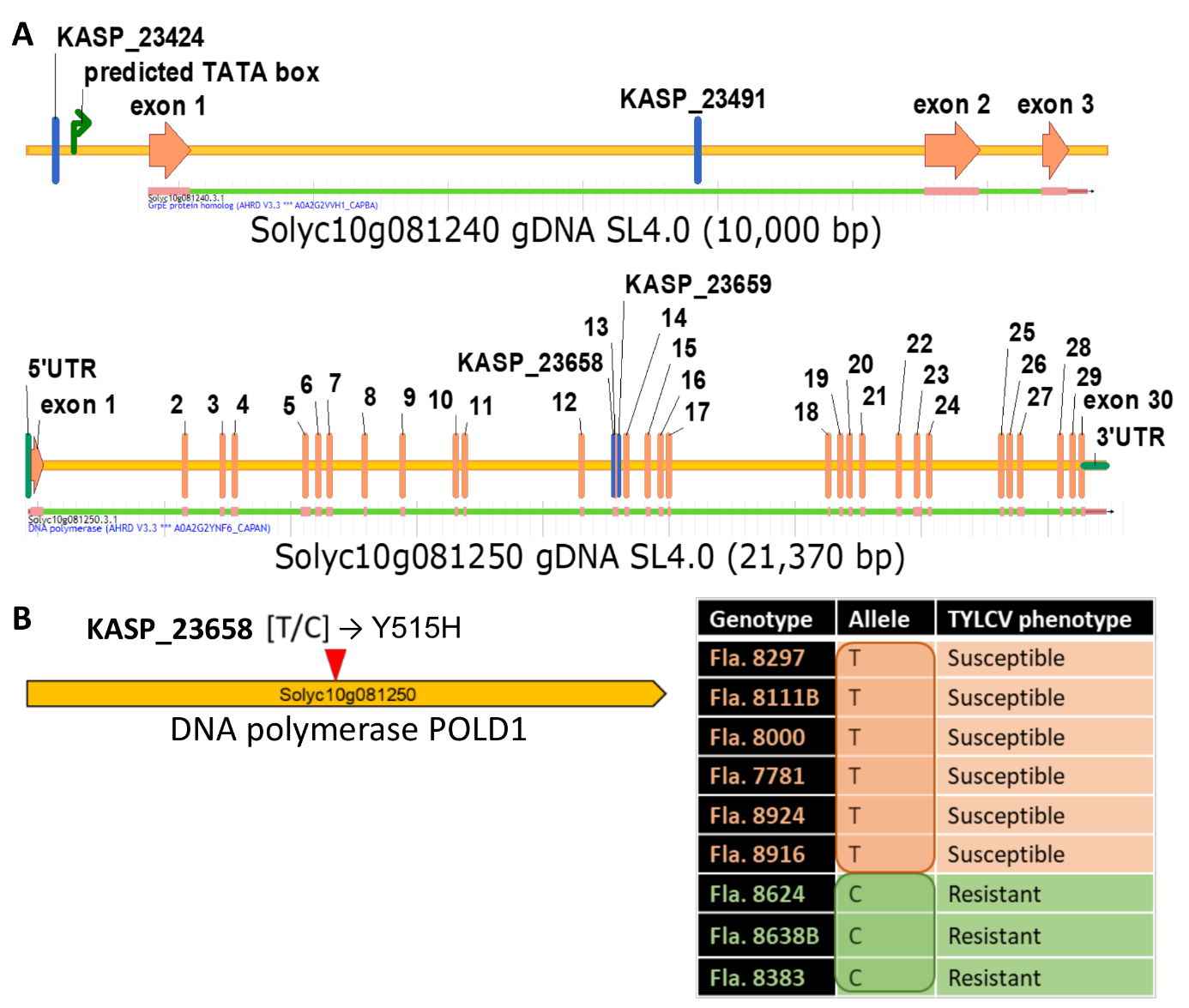


**Fig. S3** **A.** The positions of KASP markers based on single-nucleotide polymorphisms (SNPs) in the genomic DNA of the candidate genes. The orange arrows and vertical lines represent exons. The blue lines indicate the positions of the KASP markers. **B.** Correlation between allele composition for KASP_23658 SNP in exon 13 of Solyc10g081250 and TYLCV resistance.


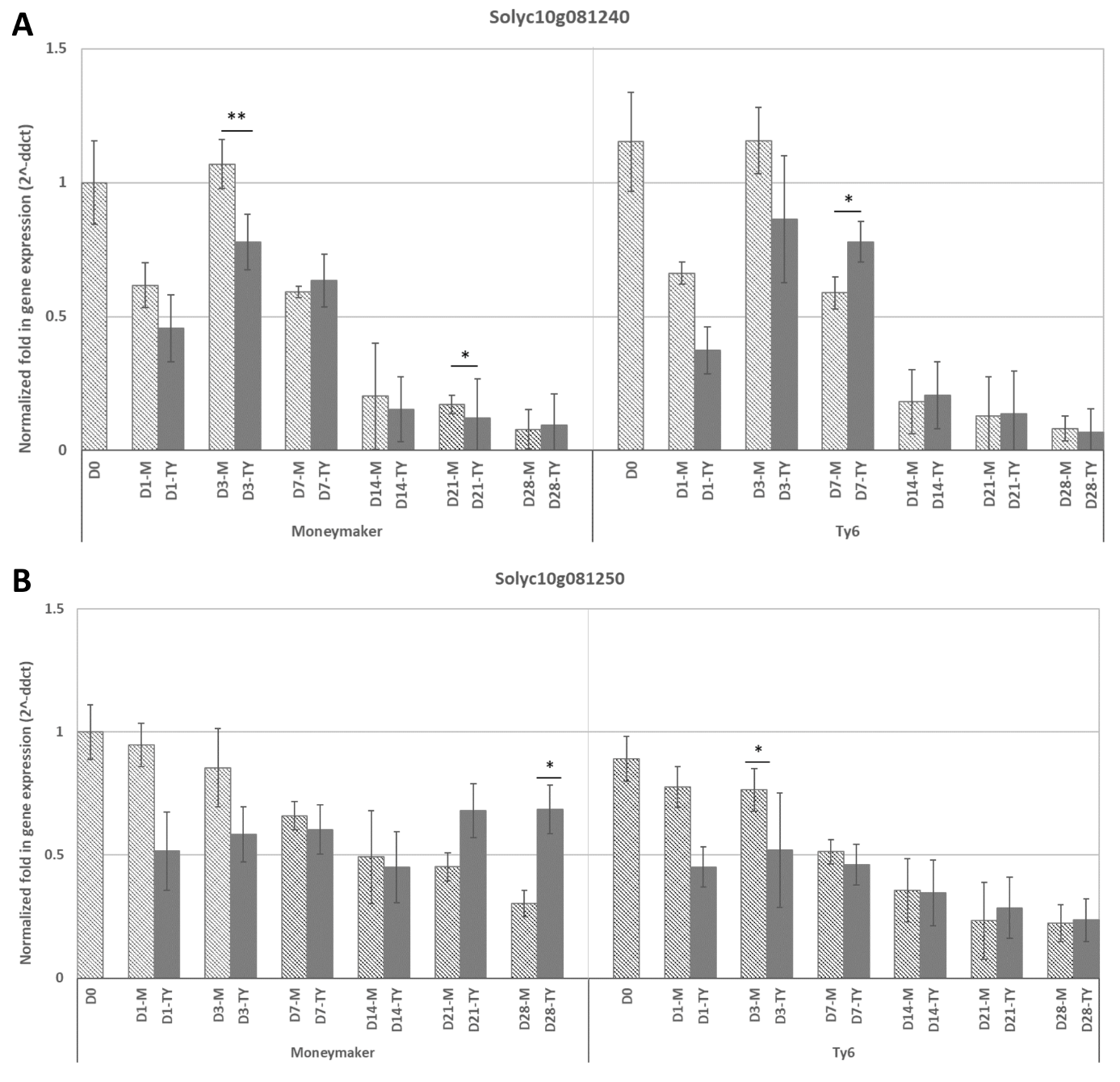


**Fig. S4** Expression level of the candidate genes in mock-treated or TYLCV-infected *Ty-6* and Moneymaker plants at different time points. **A.** Solyc10g081240. **B.** Solyc10g081250. The data represent mean values (± standard deviation) of the expression levels from three biological replicates. Values are normalized against Moneymaker time point D0 (day 0). D0, D1, D3, D7, D14, D21, and D28, samples were taken at 0, 1, 3, 7, 14, 21, or 28 days after treatment; M represents mock treatment, TY represents TYLCV infection. Light grey bars indicate mock-treated plants, dark grey bars indicate TYLCV-treated plants. *=P<0.05, **=P<0.01.


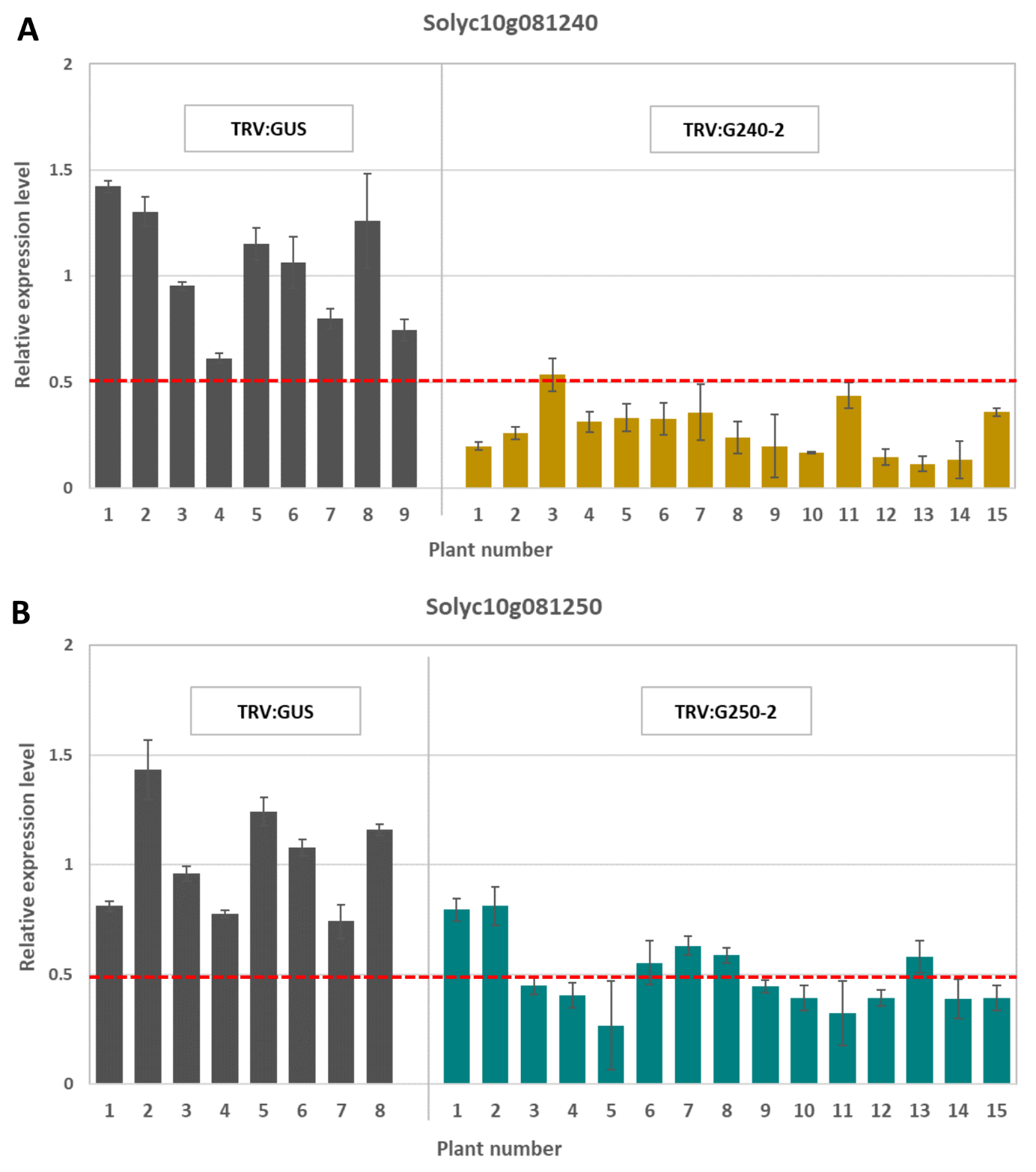


**Fig. S5** Reduced expression levels in TRV-infected *Ty-6* plants at 32 dpi in the first VIGS experiment. **A.** Solyc10g081240. **B.** Solyc10g081250. Expression values were calculated relative to the housekeeping gene *Ubiquitin* and normalized against the mean of the TRV:GUS plants. Error bars indicate standard deviation.

**
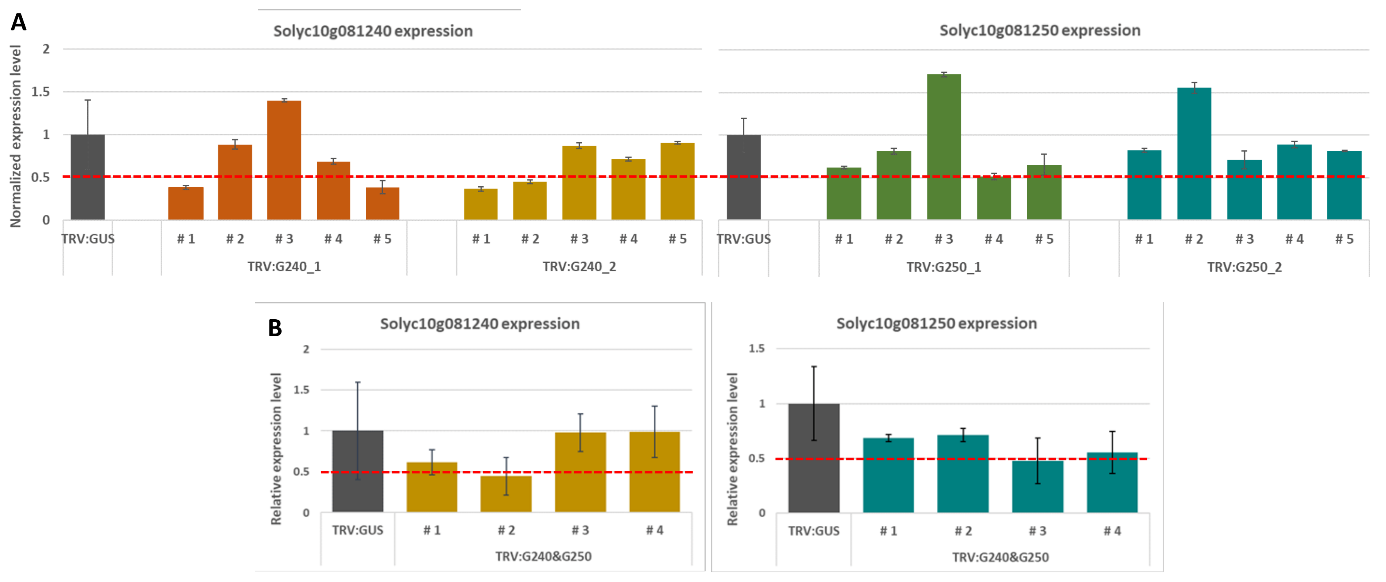
Fig. S6** Relative expression levels of candidate genes Solyc10g081240 (G240) and Solyc10g081250 (G250) in VIGS-treated *Ty-6* plants. **A.** Results of second VIGS experiment. Five individual plants were tested via qPCR for each VIGS construct. Per gene, two constructs were tested. **B.** Results of third VIGS experiment. *Ty-6* plants were co-infiltrated with TRV:G240 and TRV:G250 VIGS constructs. Expression levels were normalized to the average value of three individual TRV:GUS *Ty-6* plants. Error bars indicate standard deviation.


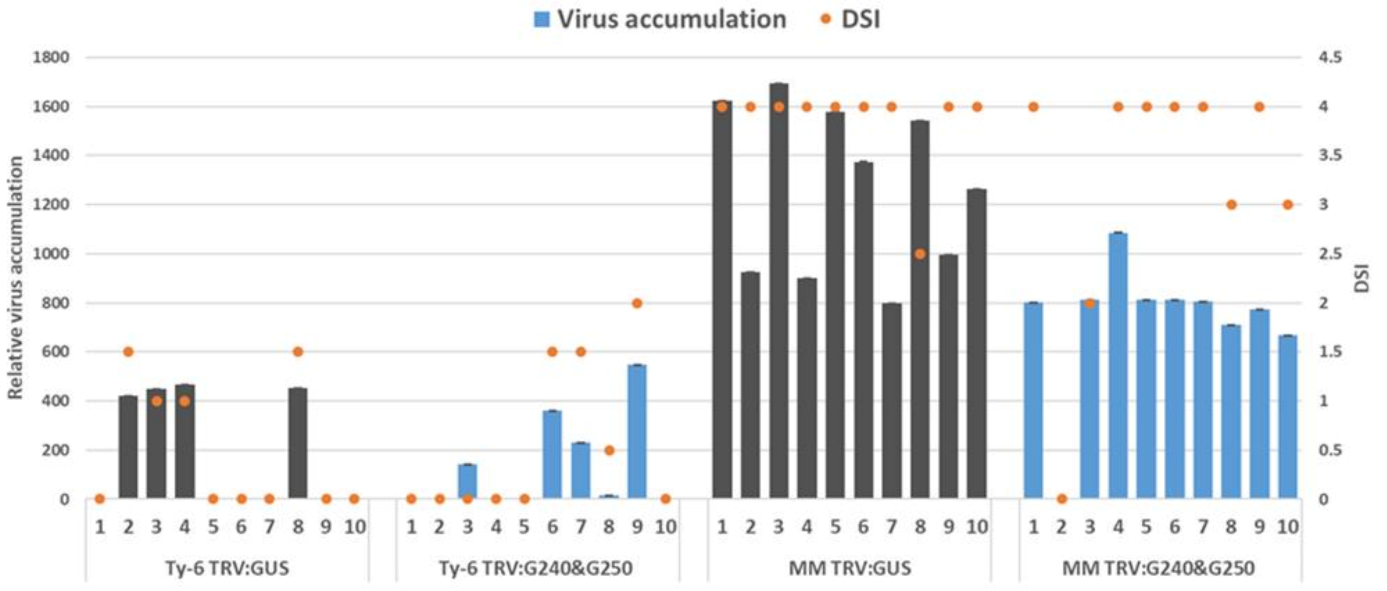


**Fig. S7** Evaluation of virus accumulation and disease severity index (DSI) in co-infiltrated *Ty-6* plants and Moneymaker (MM). TRV:GUS plants were included as a control. Expression levels were normalized to the mean of *Ty-6* TRV:GUS plants. Bar graphs indicate the virus accumulation in each plant; scatter spots indicate the DSI scores of each plant.

**
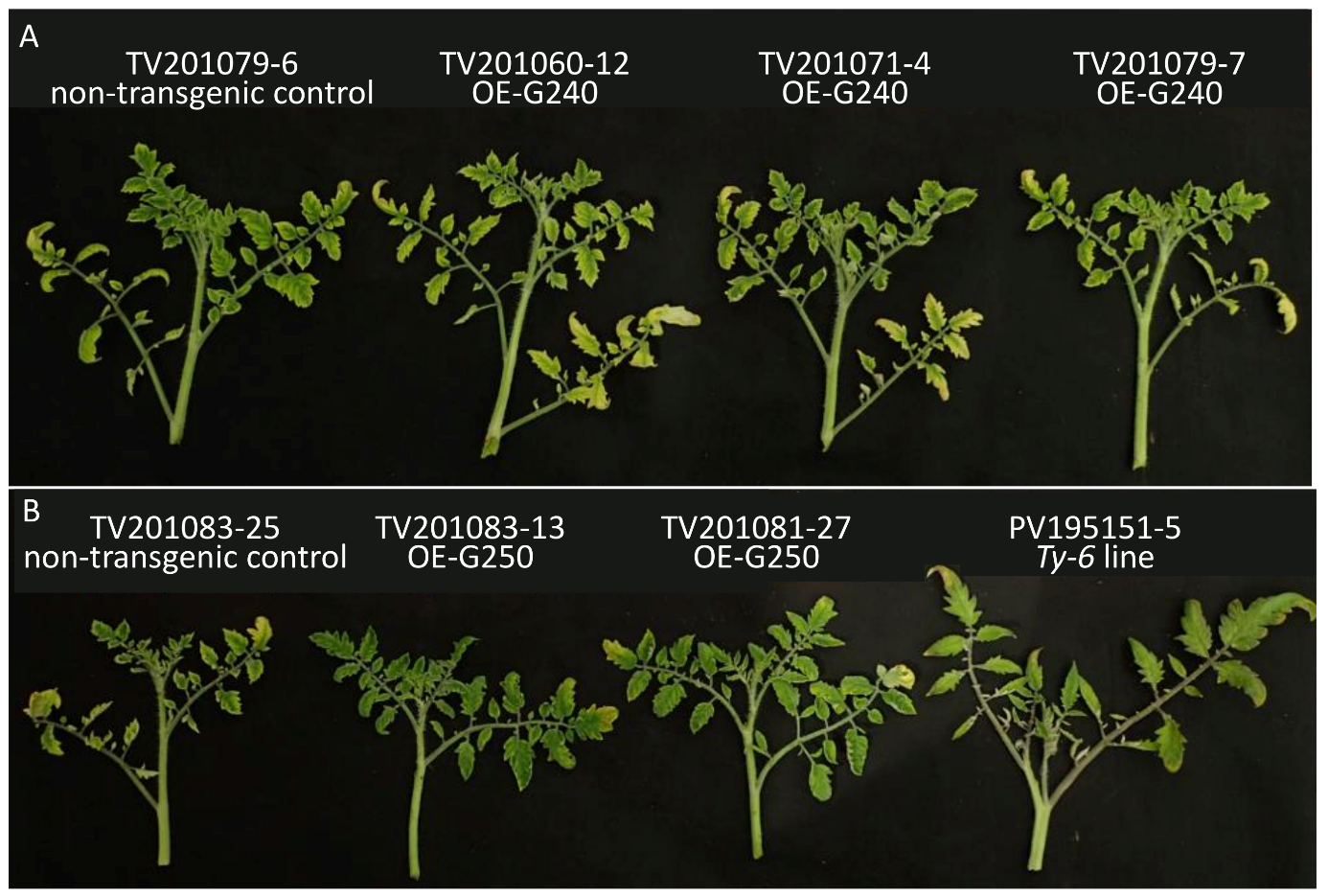
**

**Fig. S8** TYLCV symptom expression in T2 families overexpressing the *Ty-6* allele of Solyc10g081240 (panel **A**) or Solyc10g081250 (panel **B**) compared with the non-transgenic T2 plants (without 35S promotor, with TYLCV infection) at 45 dpi. TV201060, TV201071, and TV201079 are T2 plants with overexpressing the *Ty-6* allele of Solyc10g081240; TV201081 and TV201083 are T2 plants with overexpressing the *Ty-6* allele of Solyc10g081250.


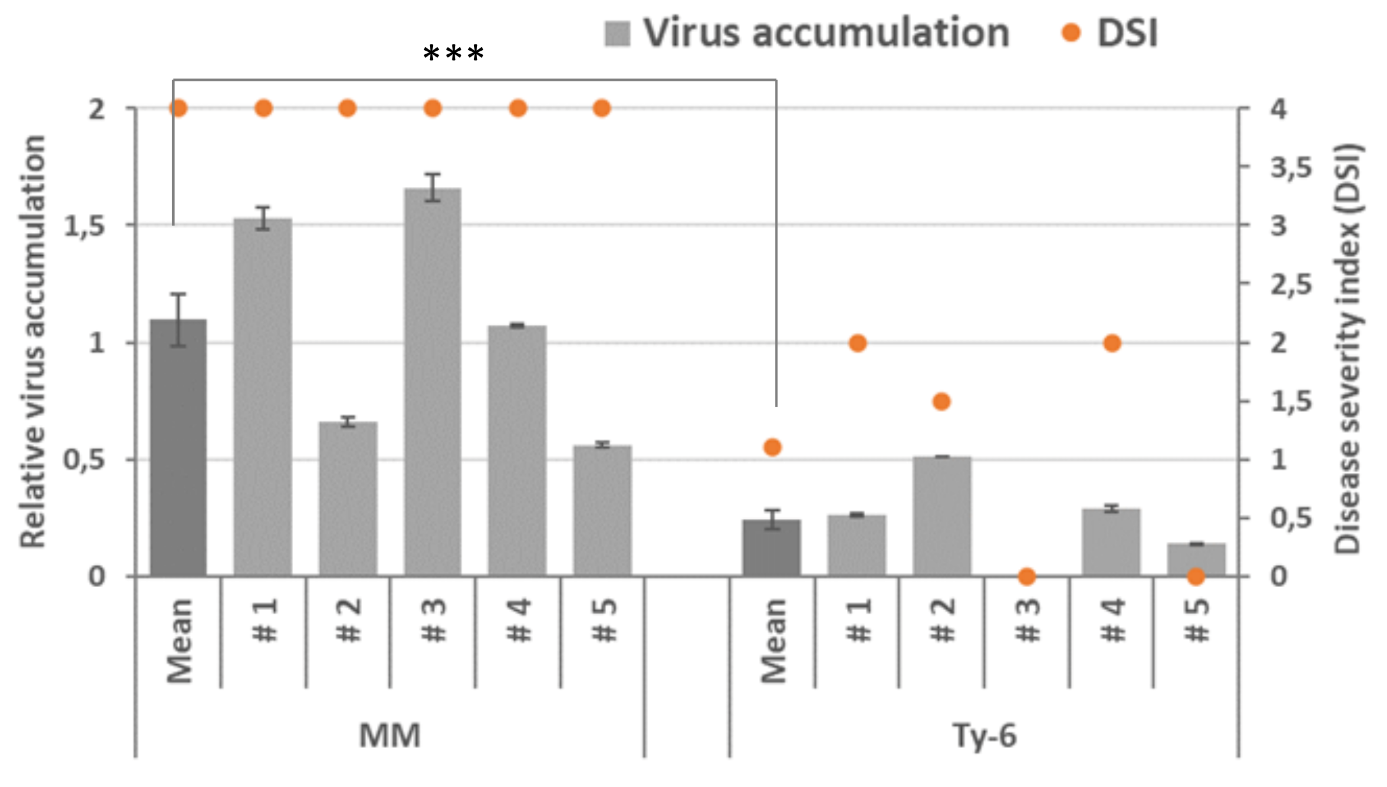


**Fig. S9** Relative TYLCV accumulation in the *Ty-6* line and MM susceptible controls were quantified via qPCR. Elongation factor 1α (*EF1*α) was used as a housekeeping gene. Error bars represent standard error. “Mean” represents average virus accumulation levels of MM and *Ty-6* line, respectively. Statistical analysis was performed on the mean values of virus accumulation (level of significance ***=p<0.001).


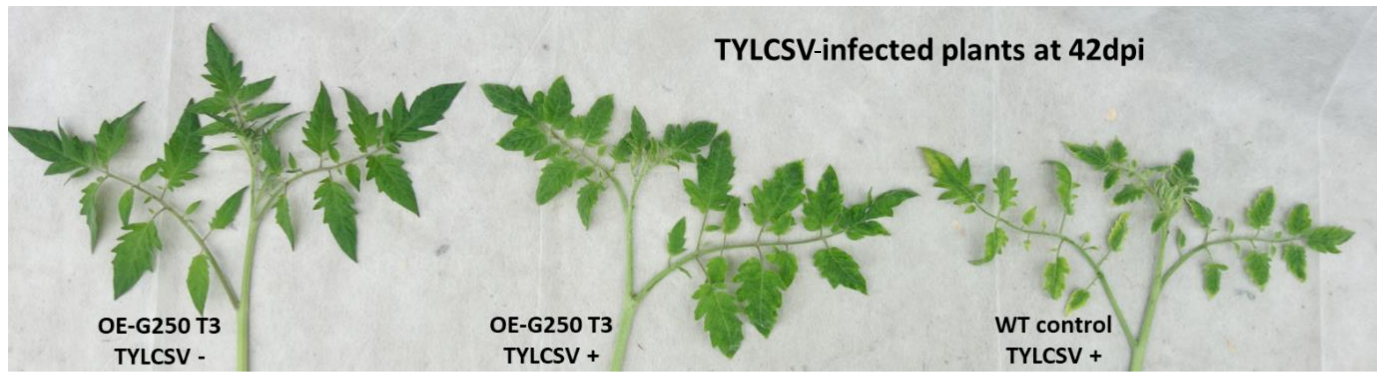


**Fig. S10** TYLCSV symptom expression in homozygous T3 plant compared to the wild-type control at 42 dpi. OE-G250 T3 means T3 generation of transgenic plant overexpression Solyc10g081250. TYLCSV+ means plants infected with TYLCSV, and TYLCSV- means plants without TYLCV infection. WT control is non-transgenic T3 plants.


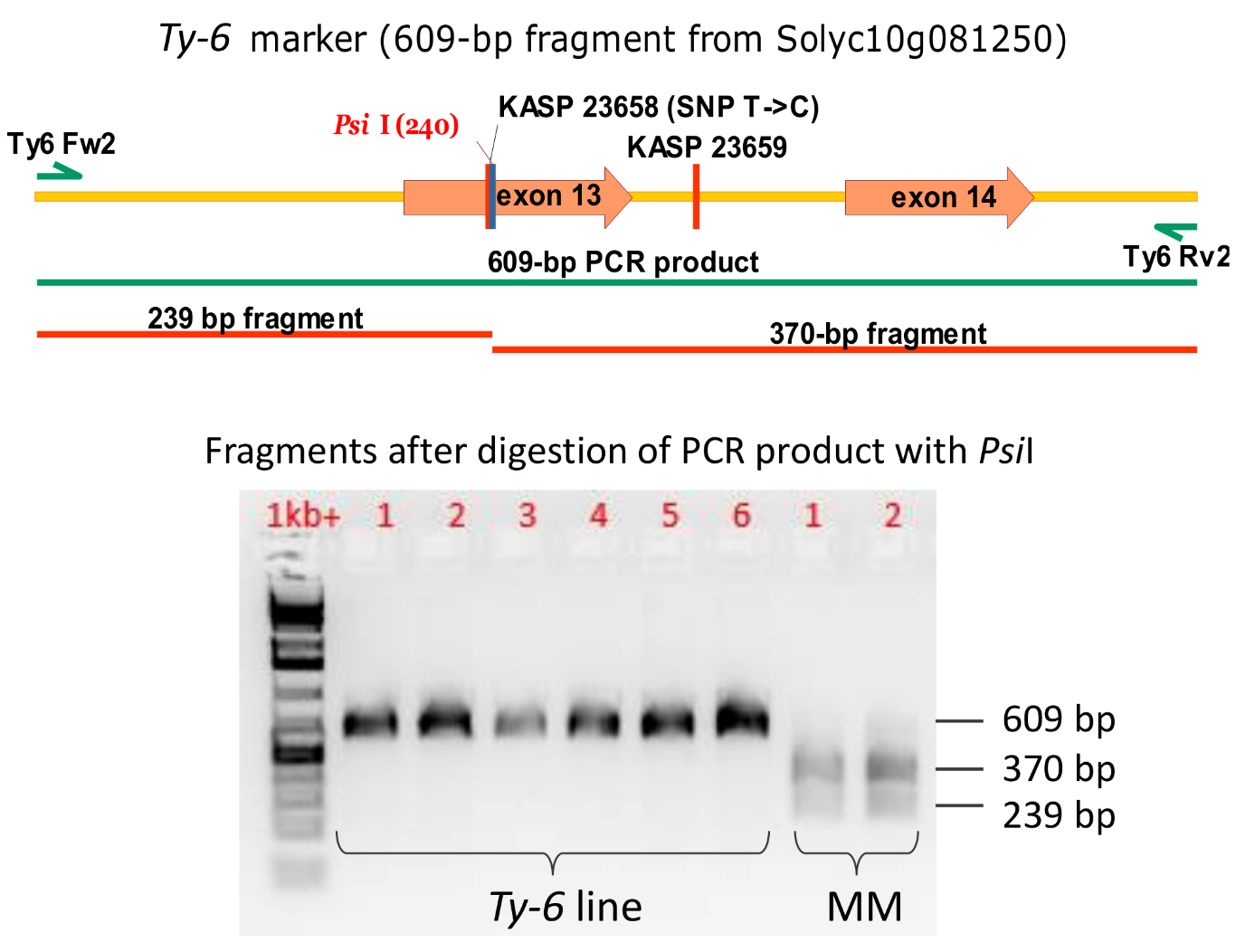


**Fig. S11** In-gene CAPS marker for *Ty-6*.

**
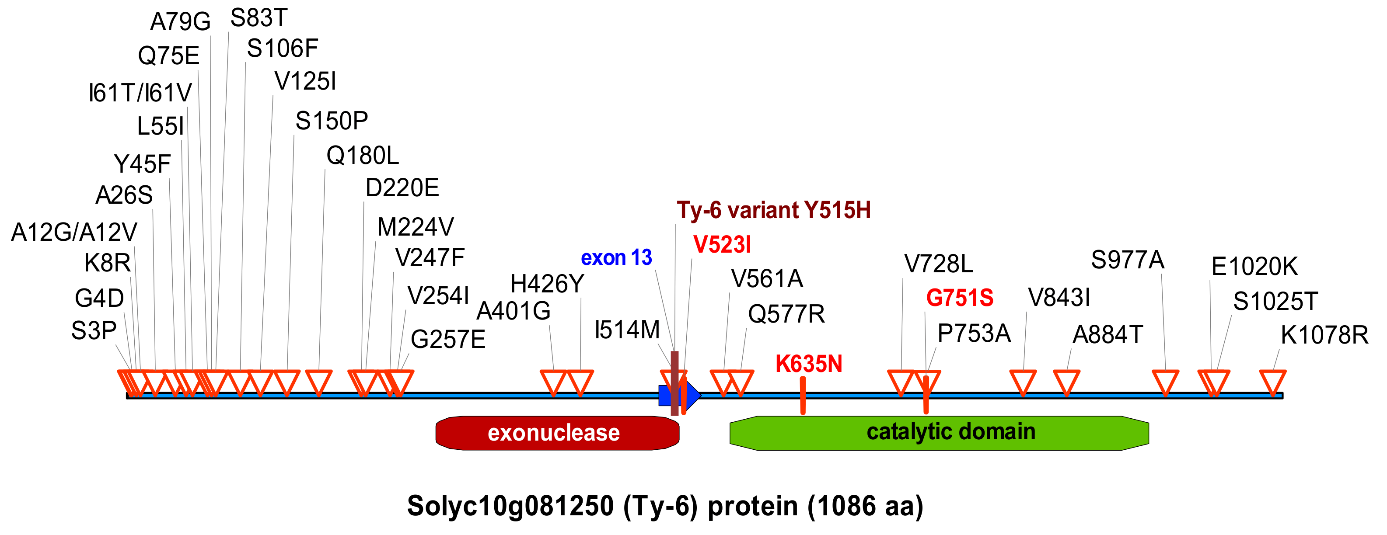
**

**Fig. S12** The distribution of nonsynonymous variants in tomato POLD1 protein sequence. Two functional domains (exonuclease and catalytic domain) were predicted using the NCBI’s CD-Search service and labeled on the map. The variants that were predicted to cause damage to protein function by two prediction programs are indicated in red.
