# Supplementary material for: The tomato gene Ty-6, encoding DNA polymerase delta subunit 1, confers broad resistance to Geminiviruses
Source: Theor Appl Genet. 2025 Jan 8;138(1):22. doi: 10.1007/s00122-024-04803-w (PMC11711579; doi:10.1007/s00122-024-04803-w)
Supplement: Supplementary file 2 — Supplementary file2 (PDF 179 kb) [file 122_2024_4803_MOESM2_ESM.pdf]

Supplementary Table S1. Markers for recombinant screening

Marker name[1]	Physical position[2]	Flanking sequence[3]	Forward primer (5'-3')	Reverse primer (5'-3')	Tomato gene	
KASP_21951	61304797	CCAAGTTTGACATAGCAATA[C/A]CAACCTAAAATAGTGAGAAT	n/a [4]	n/a	Solyc10g081010.3.1	intron
KASP_22109	61320596	CTACTCTACCTTTCAAGCCA[T/C]GGACTCGGCTAGTGGATACA	n/a	n/a	Solyc10g081010.3.1	intron
KASP_22499	61359585	CTTTGAGTTAGTGTAATTCC[T/A]TTGGACTAGACTATGTGTTG	n/a	n/a	Solyc10g081100.3.1	intron
KASP_22720	61381682	GCATCAACAATCTAATCACA[C/T]TGCAAAAACACACTTAGACA	n/a	n/a	intergenic	
KASP_22763	61386044	CCACATTTTTATTATCCACA[G/C]TAATGTGATTTTGAAACAAA	n/a	n/a	Solyc10g081130.2.1	intron
KASP_22845	61394163	CTCCGTCATCTCTTCCATCA[C/G]CCTCTCAGTCTAGCTTTGGC	n/a	n/a	intergenic	
KASP_22936	61403292	AATTTCTTTTATTTATTCTT[T/C]AAAAAAAGTATTTAAATCAA	n/a	n/a	intergenic	
KASP_22954	61405081	TCGATGTAAATTCGAGTACA[A/G]CGTTTGAAAATAAAGTATAC	n/a	n/a	intergenic	
KASP_23031	61412812	CCTCCCATCAACACAAGTAC[G/C]GGGTAACTCTATAACCGTGA	n/a	n/a	Solyc10g081170.2.1	intron
KASP_23067	61416432	ATTAATGGGATAATGTCCAA[G/C]TACCCCCTCAACTTATGCCC	n/a	n/a	intergenic	
HRM-5547-1	61434517	AGTGTAATCCCACAAGGGAA[G/T]TTTGGGGAGGGTAGAGTGTA	GTGTAATCCCACAAGGGA	TAAGGTCAGCCTACACTCTA	Solyc10g081200.1.1	intron
KASP_23424	61451371	TTATGAAGCTTTATTTTCTT[A/T]GATGAATTTTATACCAAAAT	n/a	n/a	intergenic	
KASP_23491	61457325	AAGGAGTGAGAAAGTTGCCT[G/A]GGGCTAGGTTTGATATTGTT	n/a	n/a	Solyc10g081240.3.1	intron
KASP_23658	61473934	TGGACAAATTAATGTTCATT[T/C]ATAACTATGTGGAGATGGCT	n/a	n/a	Solyc10g081250.3.1	exon
KASP_23659	61474043	TGCTTCATTTTCCTCTAGTG[T/G]AAAATTAAGTAAGGTCTCAT	n/a	n/a	Solyc10g081250.3.1	intron
HRM-6519	61504651	TCATTTTATTAGATAGTTCA[A/G]TAAAAAATATAATTTACTTT	CGAAATTTGACATTCTTT	AAATGTTTACCAAGTAAGA	intergenic	
HRM-0239	61548382	AATATGGGTGGTCTAGTGAT[C/T]GAAAAAATGGGATTGAAAAC	TAGAATATGGGTGGTCTA	TTTGTATTTGAAACCTTAGT	intergenic	
KASP_24836	61590710	GACTTTTTTGCCCTTGCTTT[C/T]TGTTTGGAAAAAATGCGGAC	n/a	n/a	Solyc10g081410.2.1	exon
KASP_25627	61669341	GTATTTGTATACAATGATTT[A/G]TATTTGTATACGACGATGAT	n/a	n/a	intergenic	
KASP_26665	61773586	TATATGAATTCAAAAAAAAA[T/A]AATAAAAATATCTTACATAAA	n/a	n/a	intergenic	
KASP_27008	61807917	GAATTCAAAATTCTAATTTC[G/A]AACATGCTTAGGTGAGTATT	n/a	n/a	intergenic	
KASP_27518	61858949	TGTCTGCATTGGCGCCTCTT[G/T]TAGGGAATGAAAGACTTTTC	n/a	n/a	Solyc10g081730.2.1	exon
KASP_27552	61862359	CCCTCAAATTATCGTACATG[G/A]TATATGTAGATAACATTCGT	n/a	n/a	intergenic	

557 Kb

174 Kb

47 Kb

[1] Kompetitive Allele Specific PCR (KASP) markers are identified by the prefix "KASP"; High Resolution Melting (HRM) markers are identified by the HRM prefix.

[2] Physical positions are based on the SL4.0 tomato genome assembly, available through the Solanaceae Genomics Network (SGH; https://www.sgn.cornell.edu/)

[3] The first allele for each SNP corresponds to the Heinz 1706 reference allele. The 2nd allele corresponds to the Ty-6 allele in all cases except for markers HRM-5547-1 and HRM-0239, in which cases the Ty-6 allele is monorphic with the reference allele but polymorphic with the susceptible parent used in the fine mapping population.

[4] All KASP markers were developed by Ag-Biotech, Inc. and are proprietary; primer sequences are unavailable.

Supplementary Table S2. Primers and sgRNAs used in the identification of candidate genes.

Gene	Name	Sequence 5'-3'	Product size (bp)	Reference
**Primers for qPCR expression analysis**				
TYLCV genome	TYLCV-IS 1678F	TTCGTCTAGATATTCCCTATATGAGGAGGTA	90	Powell et al 2012[1]
(AJ489258)	TYLCV-CONS 1756R	GGGAAGCCCATTCAAATTAAAGG		
Solyc07g064130	Ubiquitin-F	GGACGGACGTACTCTAGCTGAT	134	Yang et al. 2014[2]
(Ubiquitin)	Ubiquitin-R	AGCTTTCGACCTCAAGGGTA		
Solyc10g081240	G240-QF1	CTGAAACGGAGACGGAGACG	137	
	G240-QR1	TGAAGCTGCAACGACCGATA		
Solyc10g081250	G250-QF3	AGGAAGGCGAAGCAAAAGGA	82	
	G250-QR3	CCGTAGCCCCCTCAAATGTT		
**Primers for ampliying silencing fragements**				
Solyc10g081240	RNSol10g081240 F1	caccTTCTCCCTCCATTTCCACTG	188 (exon 1)	
VIGS/RNAi_1	RNSol10g081240 R1	ACACCGTCTGGGGAGTAATG		
Solyc10g081240	RNSol10g081240 F2	caccCGCAAAGGTTTCAGACTTGG	163 (exon 3)	
VIGS/RNAi_2	RNSol10g081240 R2	CACCTGTCCCTTCATCACCT		
Solyc10g081250	RNSol10g081250 F1	caccTCAACAGCAGCAGAAACACC	155 (exon 1)	
VIGS/RNAi_1	RNSol10g081250 R1	TCCACTTCTGAAGCCTCTCC		
Solyc10g081250	RNSol10g081250 F2	caccTATCGGAGGAAGAAGGCTCA	162 (exon 30)	
VIGS/RNAi_2	RNSol10g081250 R2	TTATGCTGCTTCCACACCAG		
**Primers for checking the presence of the transgenic insert**				
NPTII	NPTIIAf_351	TGCTCGACGTTGTCACTGAA	351	
	NPTIIAr_351	TGATATTCGGCAAGCAGGCA		
CaMV 35S promoter	35S_Fw	TACAAAGGCGGCAACAAACG	597	
(pHELLSGATE8)	35S_Rev	AGCAAGCCTTGAATCGTCCA		
CaMV 35S promoter	PK7WG2-35SF	TGTGCGTCATCCCTTACGTC	551	
(Pk7WG2)	PK7WG2-35SR	TGGACGATTCAAGGCTTGCT		
**sgRNAs for Crispr/Cas9 constructs**				
	sgRNA1	CCGTCAGCGCCACCGCTTAC		
Solyc10g081240	sgRNA2	CAGACGGTGTCGTATTCACC		
(Construct CC:G240)	sgRNA3	CTCCGCTAACTCAATTGTGC		
	sgRNA4	GCAGGTAGAAGAGTCCGAGC		
	sgRNA1	CAACTCTAAGAAGCGATCGG		
Solyc10g081250	sgRNA2	GCAGAAACACCACGCAGCCA		
(Construct CC:G250)	sgRNA3	GGAAGAAGAGGACCTAGACG		
	sgRNA4	AGAGCAATTCCATAAAGGAG		
**Primers for detecting mutagensis**				
Solyc10g081240	G240_TR check F1	TTCTCCCTCCATTTCCACTG	412	
	G240_TR check R1	CCCAACAAATTCAGCCATAAA		
Solyc10g081250	G250_TR check F1	AACCCTCTTCTCCCAACTCC	340	
	G240_TR check R1	GAAGGCATGAAGTCGGAAAT		
**Primers for cloning coding sequence of the *Ty-6* candidate genes**				
Solyc10g081240	G240-pD221-attB1	GGGGACAAGTTTgtacaaaaaagcaggctAT**ATG**TTTGAGAATCTTCTCAAAGAACC		
	G240-pD221-attB2	GGGGACCACTTTGTACAAGaaagctgggtA**TTA**GTTACCTCCCTCACCTGTCC		
Solyc10g081250	G250-pD221-attB1	GGGGACAAGTTTgtacaaaaaagcaggctAT**ATG**AACTCCGGCAACTCTAAG		
	G250-pD221-attB2	GGGGACCACTTTGTACAAGaaagctgggtA**TTA**AAAGTTCCACCGATCTAATTG		
**Primers for in-gene *Ty-6* marker**				
Solyc10g081250	Ty6_Fw2	CTGTCATCCTTTCCCAAATACA	609	this manuscript
	Ty6_Rv2	TGGCACAACAGGAGCTTTACAA	digest with *Psi* I	

[1]Powell, M. E., Cuthbertson, A. G. S., Bell, H. A., Boonham, N., Morris, J., & Northing, P. (2012). First record of the Q Biotype of the sweetpotato whitefly, Bemisia tabaci, intercepted in the UK. *European Journal of Plant Pathology* , 133(4), 797–801. https://doi.org/10.1007/s10658-012-9976-6

[2]Yang, X., Caro, M., Hutton, S. F., Scott, J. W., Guo, Y., Wang, X., Rashid, M. H., Szinay, D., de Jong, H., Visser, R. G. F., Bai, Y., & Du, Y. (2014). Fine mapping of the tomato yellow leaf curl virus resistance gene *Ty-2* on chromosome 11 of tomato. *Molecular Breeding* , 34(2), 749–760. https://doi.org/10.1007/s11032-014-0072-9

Supplementary Table S3. The effects of knocking down/out the candidate genes on regeneration in vitro.

Construct[1]	Regeneration efficiency	Shoot formation	Phenotype
RNAi:G240-2	37%		
RNAi:G250-2	18%	Most calli generate only a single leaf, no shoots	Light green and white leaves
CC:G240	10%		
CC:G250	17%		

[1]RNAi, silencing construct; CC, CRISPR/Cas9 construct; G240, Solyc10g081240; G250, Solyc10g081250

Supplementary Table S4. List of T2 transformants overexpressing Solyc10g081250 (OE-G250) used for producing T3 generation.

T1	T2	Expression level	Relative virus accumulation[2]	DSI	#Seeds	T3
**TV201081**	TV201081-8	8.61	1.63	2	150	TV212065
	TV201081-10	15.98	1.31	2	150	TV212064
	TV201081-12	14.57	2.21	2	100	TV212063
	TV201081-15	15.63	1.71	2	150	TV212062
	TV201081-18	17.98	0.65	2	150	TV212061[3]
	TV201081-19	15.95	0.91	2	100	TV212060[3]
	TV201081-21[1]	1.54	3.92	3.5	100	TV212059
**TV201083**	TV201083-2[1]	1.27	6.89	3.5	25	TV212058
	TV201083-9	16.71	1.25	2	150	TV212057[3]
	TV201083-10	4.77	1.50	2	150	TV212056
	TV201083-14	6.91	4.10	2	150	TV212055
	TV201083-19	5.36	2.92	2.5	25	TV212054
	TV201083-20	3.71	1.77	2	150	TV212053
	TV201083-21	7.19	3.14	2	70	TV212052

Note: the expression level, virus accumulation, and disease severity index (DSI) data are from the first TYLCV disease assays on T2 plants.

[1] TV201081-21 and TV201083-2 are non-transgenic plants.

[2] Relative virus accumulation compared with level in *Ty-6* plants

[3] TV212061, TV212060 and TV212057 are T3 families used for disease assay

Supplementary Table S5. Germination of T3 transformants overexpressing Solyc10g081250 (OE-G250), and test for the presence of transgene using PCR with 35S promoter primers

T3 family	#Seeds sown	#Germinated	#Plants with 35S promoter
TV212057[1]	60	24	24
TV212060	60	53	53
TV212061	60	54	54

[1]Approximately 15 additional seeds germinated late, and presence of 35S was not tested on these seedlings

Supplementary Table S6. Non-synonymous SNPs present in Solyc10g081250 allelic variations.

	150 variants	amino acid position																																			
**Accession number**	**Accession name**	**3**	**4**	**8**	**12**	**12**	**26**	**45**	**55**	**61**	**75**	**79**	**83**	**106**	**125**	**150**	**180**	**220**	**224**	**247**	**254**	**257**	**401**	**426**	**514**	**515**	**523**	**561**	**577**	**635**	**728**	**751**	**753**	**843**	**884**	**977**	**1020**	**1025**	**1078**
3	Solanum lycopersicum Gardeners Delight																											V561A											
7	Solanum lycopersicum Katinka Cherry																											V561A											
17	Solanum lycopersicum LYC3340																											V561A											
22	Solanum lycopersicum PI129097																											V561A											
39	Solanum lycopersicum LA0113																											V561A											
42	Solanum lycopersicum LYC2962																											V561A											
45	Solanum lycopersicum LYC2740																											V561A											
105	**Solanum lycopersicum LA1479**	S3P		K8R																							**V523I**												
25	Solanum corneliomuelleri LA0118							Y45F																					Q577R				P753A		A884T	S977A			
44	Solanum pimpinellifolium LYC2798																											V561A											
46	Solanum pimpinellifolium LA1584																											V561A											
47	Solanum pimpinellifolium LA1578	S3P																										V561A											
49	Solanum peruvianum LA1278							Y45F																				V561A	Q577R									S1025T	
60	Solanum peruvianum LA1954		G4D							I61V												G257E							Q577R				P753A		A884T	S977A		S1025T	
51	Solanum chmielewskii LA2663											A79G																V561A		K635N									
52	Solanum chmielewskii LA2695											A79G																V561A		K635N									
53	Solanum galapagense LA0483										Q75E																												
104	Solanum galapagense LA1044										Q75E																												
56	Solanum neorickii LA2133						A26S										Q180L											V561A											
57	Solanum neorickii CGN24193												S83T				Q180L											V561A											
58	Solanum arcanum LA2157															S150P		D220E										V561A											
59	Solanum arcanum LA2172																Q180L											V561A											
62	Solanum huaylense LA1983																											V561A											
63	Solanum huaylense LA1365							Y45F															A401G		I514M				Q577R				P753A	V843I	A884T	S977A		S1025T	
75	Solanum huaylense LA1364							Y45F											M224V				A401G		I514M			V561A	Q577R		V728L						E1020K	S1025T	
64	Solanum chilense CGN15532							Y45F																					Q577R				P753A		A884T	S977A		S1025T	
65	Solanum chilense CGN15530									I61V											V254I								Q577R				P753A		A884T	S977A		S1025T	
66	Solanum habrochaites f. glabratum CGN15791				A12V	A12G														V247F				H426Y				V561A	Q577R			G751S				S977A			
67	Solanum habrochaites f. glabratum PI134418																			V247F				H426Y				V561A	Q577R			G751S				S977A			
68	Solanum habrochaites f. glabratum CGN15792				A12V															V247F				H426Y				V561A	Q577R			G751S				S977A			
69	Solanum habrochaites f. glabratum LA1718								L55I	I61T					V125I					V247F				H426Y				V561A	Q577R			G751S		V843I		S977A			K1078R
70	Solanum habrochaites LA1777																											V561A	Q577R			G751S		V843I		S977A			
71	Solanum habrochaites f. glabratum LA407				A12V															V247F				H426Y				V561A	Q577R			G751S				S977A			
72	Solanum habrochaites LYC4				A12V															V247F				H426Y				V561A	Q577R			G751S				S977A			
74	Solanum pennellii LA716													S106F														V561A								S977A			
This study	***Ty-6* line**																									**Y515H**													

Note: **1**. Variants that may impact the protein function according to the SNP effect prediction programs are in red and orange color; **2**. V523I locates close to Y515H; **3**. K635N and G751S are likely species-specific.
